# Computational Paradigm to Elucidate the Effects of Arts-Based Approaches and Interventions: Individual and Collective Emerging Behaviors in Artwork Construction

**DOI:** 10.1371/journal.pone.0126467

**Published:** 2015-06-10

**Authors:** Billie Sandak, Ephrat Huss, Orly Sarid, David Harel

**Affiliations:** 1 Department of Computer Science and Applied Mathematics, Faculty of Mathematics and Computer Science, The Weizmann Institute of Science, Rehovot, Israel; 2 The Spitzer Department of Social Work, Faculty of Humanities & Social Sciences, Ben-Gurion University of the Negev, Beer Sheva, Israel; University of Westminster, UNITED KINGDOM

## Abstract

Art therapy, as well as other arts-based therapies and interventions, is used to reduce pain, stress, depression, breathlessness and other symptoms in a wide variety of serious and chronic diseases, such as cancer, Alzheimer and schizophrenia. Arts-based approaches are also known to contribute to one’s well-being and quality of life. However, much research is required, since the mechanisms by which these non-pharmacological treatments exert their therapeutic and psychosocial effects are not adequately understood. A typical clinical setting utilizing the arts consists of the creation work itself, such as the artwork, as well as the therapist and the patient, all of which constitute a rich and dynamic environment of occurrences. The underlying complex, simultaneous and interwoven processes of this setting are often considered intractable to human observers, and as a consequence are usually interpreted subjectively and described verbally, which affect their subsequent analyses and understanding. We introduce a computational research method for elucidating and analyzing emergent expressive and social behaviors, aiming to understand how arts-based approaches operate. Our methodology, which centers on the visual language of Statecharts and tools for its execution, enables rigorous qualitative and quantitative tracking, analysis and documentation of the underlying creation and interaction processes. Also, it enables one to carry out exploratory, hypotheses-generating and knowledge discovery investigations, which are empirical-based. Furthermore, we illustrate our method’s use in a proof-of-principle study, applying it to a real-world artwork investigation with human participants. We explore individual and collective emergent behaviors impacted by diverse drawing tasks, yielding significant gender and age hypotheses, which may account for variation factors in response to art use. We also discuss how to gear our research method to systematic and mechanistic investigations, as we wish to provide a broad empirical evidence for the uptake of arts-based approaches, also aiming to ameliorate their use in clinical settings.

## Introduction

Arts-based therapies and interventions are used in diverse populations and age groups to help alleviate symptoms and induce therapeutic and psychosocial effects in a wide variety of serious chronic conditions, illnesses, mental disorders, etc. For example, pain, breathlessness, stiffness, stress, depression, fatigue, anxiety and other symptoms are mitigated by the use of art therapy [1–7], music therapy [8–14], dance/movement therapy [15–17], and other arts-based approaches and interventions [18], in a wide variety of serious and chronic diseases, such as cancer, cardiovascular, dementias, infectious and neurologic. For either a patient or a healthy individual, the engagement with the arts also enhances one’s well-being and quality of life [19]; e.g., among professionals [20, 21]. The benefits of the use of arts are also manifested in psychophysiological measurements; e.g., reduction of cortisol levels and blood pressure [22–27]. Although arts-based therapy has been employed clinically for more than a century, in hospitals, community centers, education facilities, etc. [28], and has been recognized as a profession for more than twenty years [29], much research is required to reveal the underlying mechanisms by which such arts-based approaches operate, and to enhance their effectivity [30–34].

A typical clinical setting utilizing the arts consists of the creation work itself, such as artwork, musical work, and dance/movement work, the therapist and the patient, a three-way relationship and entities therein, all of which constitute a rich and dynamic environment of occurrences. As such, the visual, auditory and bodily information in the modalities of the evolving artistic construction processes are difficult to grasp. For example, in artwork, these include the starting and stopping of a drawing stroke, the length, direction and velocity of that stroke, color and tool choices, erasures, etc. In musical work, these include the beginning and end of a played musical note, the pitch, intensity, tempo and instrument choices. In dance work, these include the temporal positioning of the limbs in 3D space, torso direction, turns, etc. The artistic construction work itself is not the sole component of the relevant dynamic processes; complex social interaction also occurs. A session involves the therapist and patient, their verbal and non-verbal communication, body language, bodily postures, movements, hands positioning, facial expressions, positions in the room, therapist intervention, etc. These complex, simultaneous, and interwoven expressive and social behavioral processes are often considered intractable to human observers, and as a consequence are usually interpreted subjectively and described verbally, thus affecting their subsequent analyses and understanding.

In approaches that utilize the art modality for treatment, the process of *creating* the artwork is evaluated and rated manually via self-reports, questionnaires and forms [35–39], if at all (e.g., focusing mostly on the finished artwork). Attempts have indeed been made to computerize partial information and post evaluation of the end product [40], but they do not deal with the dynamic process of its construction. Hence, in additional to psychophysiological measurements studies [41], understanding the underlying mechanistic behavioral processes of art-based approaches *in action* is required. This is true, in particular, of the quantitative identification of 'moments of change' or 'turning points' in the clinical session, their causality and measurements of change processes [32]. Art-based approaches can be carried out along the continuum of ‘art as therapy’ ↔ ‘art in therapy’ [42–44]. The former notion assumes that the art making is the therapeutic process itself and thus, the artwork is the focus of attention. In the latter, the therapist guides and intervenes, trying to initiate changes; e.g., utilizing guided imagery (a cognitive behavioral intervention [45]), and hence, the therapist-patient interaction is also considered a focus of attention.

We develop a broad *computational paradigm (CP)* for capturing, and then for empirically elucidating and analyzing, complex dynamic behavioral processes in the expressive arts, and apply it in real-world experimentation. Our technology, which centers on modeling the dynamics in the visual language of Statecharts [46] is designed to allow rigorous and quantitative tracking of the underlying creation and interaction processes, portraying expressive and social behaviors, and their objective analysis and documentation. This includes defining and examining individual and collective metrics and measurements for performance analysis and comparisons. All these allow our method to be used in investigations along the ‘art as therapy’ ↔ ‘art in therapy’ continuum providing novel insights and empirical probing abilities. Eventually it will also be used in evaluating and predicting treatment progress and outcome.

Here, we present our CP and apply an initial version thereof to a proof-of-principle artwork study with human participants. The artwork focus is the first step prior to the exploration of the contribution of therapist-patient interaction. Our study, carried out with healthy/normal subjects [47], focuses on the art making and the artistic creation/construction dynamics therein. We were able to show our method's use in the investigation of individual and collective emergent behaviors; i.e., arising properties and patterns of the decoded behavioral processes in response to several drawing tasks. We further statistically analyzed these behaviors for differences of demographic attributes in response to engagement with art making, obtaining gender- and age- significant results, factors that may account for collective variation impacted by art use.

**Fig 1:**
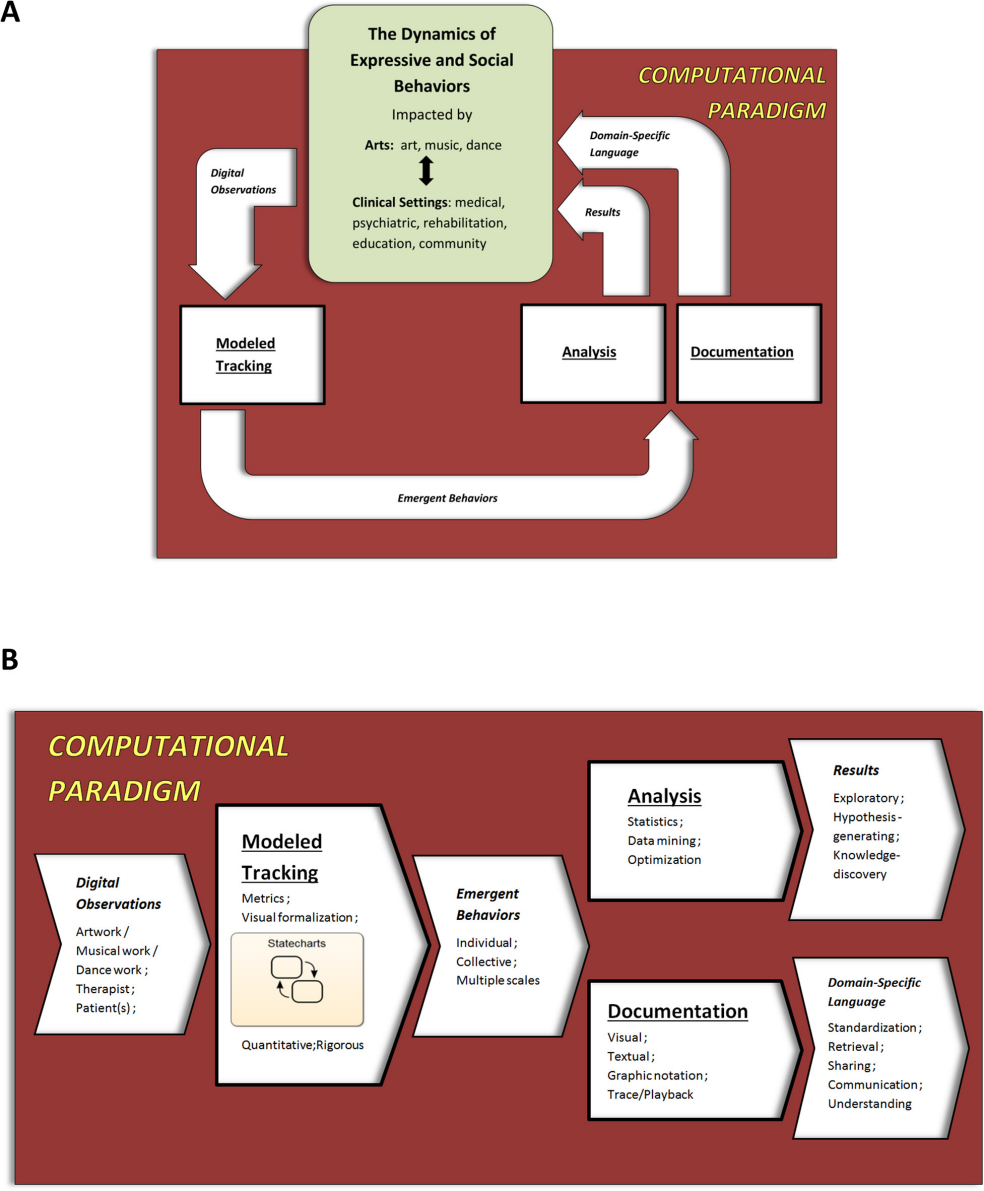
The methodology’s architecture. (**A**) The Computational Paradigm's and its comprising components. (**B**) The Computational Paradigm’s design of its processing modules and information flow.

Our Statecharts-based computational approach is designed to be useful for modeling not only the artwork, as we illustrate here, but also musical work and dance work, as well as their use in clinical setting models involving the therapist and patient. Specifically, as depicted in Fig 1A, our technological suite consists of: (i) the ***Modeled Tracking*** module, responsible for capturing the dynamics of the systems modeled, (e.g. the artwork, patient), input by digital means, (e.g., tablet touchscreens, video and audio); (ii) the ***Analysis*** module, responsible for investigating emerging individual and collective behaviors "teased out" by the use of art; (iii) the ***Documentation*** module, which transforms the expressive and social emergent behaviors into a format imaging the arts session’s occurrences.

Our CP is an enabling technology being developed to obtain novel insights and probing abilities into the mechanisms of behavioral processes impacted by the expressive arts. By developing a methodology that enables us to better understand how the engagement with the arts empirically "behaves", rather than how it is interpreted verbally and qualitatively, novel research of arts induced effects is made plausible, as described next. In the section ‘The Computational Paradigm’ we describe in detail our modeling- and analysis-based methodology. In the subsequent section, ‘Proof-of-Principle Artwork Study’ we show the method’s use of the artwork model application in a real-world art-making study with human subjects.

## The Computational Paradigm

### Overview of the Methodology’s Architecture

Our methodology’s suite is devised to enable the rigorous and quantitative tracking, analysis and documentation of artistic expression and social behavioral processes, and also allow a flexible and diversified approach for designing studies investigating these processes. As seen in Fig 1A and 1B, and briefly described earlier, this is achieved by the enabling technology's design comprised of three modules, the Modeled Tracking, Analysis and Documentation modules and the information that flows downstream between them.

Since the naked human eye cannot rigorously and objectively capture and document the observed behaviors of the system studied, we are using digital means to do so for the initial raw data, e.g., tablet touch screens and later on video and audio. The digital information is then decoded and fed into the tracking component of our paradigm, The Modeled Tracking module, which hosts the Statecharts-based model of the explored system and its specified metrics. For example, the model of the artwork system and its defined parameters/metrics, described in detail in the next sections.

The Modeled Tracking module generates quantitative emergent individual and collectives behaviors that are the input to the Analysis module, which is study-based. In this module we employ mathematical, computational and algorithmic tools, to investigate the data obtained, as dictated by the study’s aims. Our proof-of-principle artwork investigations with human participants exemplify this.

The emergent behaviors obtained are also output using our Documentation module which is developed for portraying the dynamics of the processes explored. This is done by composing textual and visual reports, as well as graphical charts, to convey the properties of the dynamics of the creation processes given in an accurate and non-verbal manner, which is easy to perceive. Eventually, we aim to develop a domain-specific language as described in the Discussion section. Furthermore, digital recordings of the dynamics allow the processes to be played back and reproduced for repetitive inspections.

Please note that the terms “creation” and “artistic construction” that define the dynamic process of expressive arts making are interchangeable, and neither has any relevance to the terms “creative” and “creativity”. Furthermore, the terms “therapist” and “clinician” are interchangeable as are the terms “patient” and “client”.

### Modeling Considerations

We apply our method to studying the construction process for art modality, as part of a broader endeavor of modeling creation processes in other modalities, such as music and dance/movement, and modeling interaction processes in the clinical setting of the arts room. Three major entities comprise the arts room: the creation work (e.g., artwork, musical work or dance work), the patient and the therapist. These components and their interactions constitute a dynamic system that continuously reacts to internal and external stimuli. Internal stimuli are exemplified by the ongoing art creation and the patient's choices therein. An example of an external stimulus is the therapist’s intervention. Such systems have been termed *reactive* [48].

Within the reactive system of the arts room, the creation/construction work itself is considered a reactive sub-system driven by stimuli of events. Such events include picking up a drawing medium, such as a paint brush or a red colored pastel crayon, starting to draw a stroke and stopping. These events transfer the system from state to state, for example, 'materials being selected' to 'artwork being worked on'. The state of 'artwork not being worked on' can be due to idleness of the *creator*; e.g., he/she is busy thinking of the next drawing step or taking a rest. We refer to the creator as anyone carrying out an art work; e.g., a patient, an artist, a painter, or a person with no art training at all. However, in a clinical setting, this latter state can also be reached when the therapist asks the client to stop drawing or when the session ends. In music, an example of an event that causes a transition from state to state is pressing the pedal of the piano, transferring the musical intensity from pianissimo (very soft) to mezzo forte (slightly loud). The tempo, for example, can change from a lento state (slow) to a state of allegro (fast) by the event of the therapist’s intervention or the creator’s choice. In dance work, a dancer or a therapy patient can be in a state where counterclockwise turns are being carried out or in one where a hand is lifted, or in both. In the event of five seconds elapsing, lying on the floor is a possible state, choreographed or intervened by the therapist.

**Fig 2:**
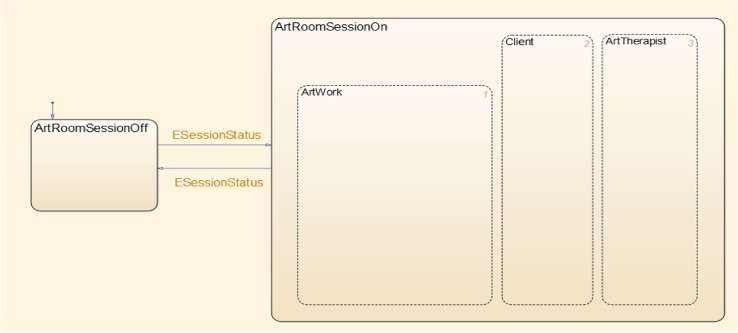
The top-level model of the system. The Statecharts visual formalism [46] exemplified in modeling the high-level state of the art room and three concurrent/orthogonal states (dashed lines) specifying the entities therein: the **Artwork**, **Client** (patient) and **ArtTherapist**. The figure also shows the events that trigger the beginning of the therapy session and its termination, specified as mutually exclusive states, **ArtRoomSessionOn** and **ArtRoomSessionOff**, respectively (with solid lines).

The state/event approach, i.e., the use of states and the events that trigger transitions between the states, is an idea that has been used since the earliest days of describing computation. It was given a boost in the 1980s with the advent of *Statecharts* [46] for specifying the behavior of complex reactive systems. Statecharts are a *visual formalism* [49], which enriches the basic state/event approach with means for describing *hierarchy* (nested states) and multi-level transitions, as well as *orthogonality* (concurrent states), and more. See Fig 2 for an example of a statechart that captures the high-level states for the art room. Statecharts constitute an intuitive, yet mathematically rigorous formalism, with broad applications in software and systems engineering, healthcare processes, automotive and aerospace, biological modeling, etc. See, e.g., [50–54]. We base our modeling method on Statecharts and its underlying execution and analysis tools.

### The System Models

**Fig 3:**
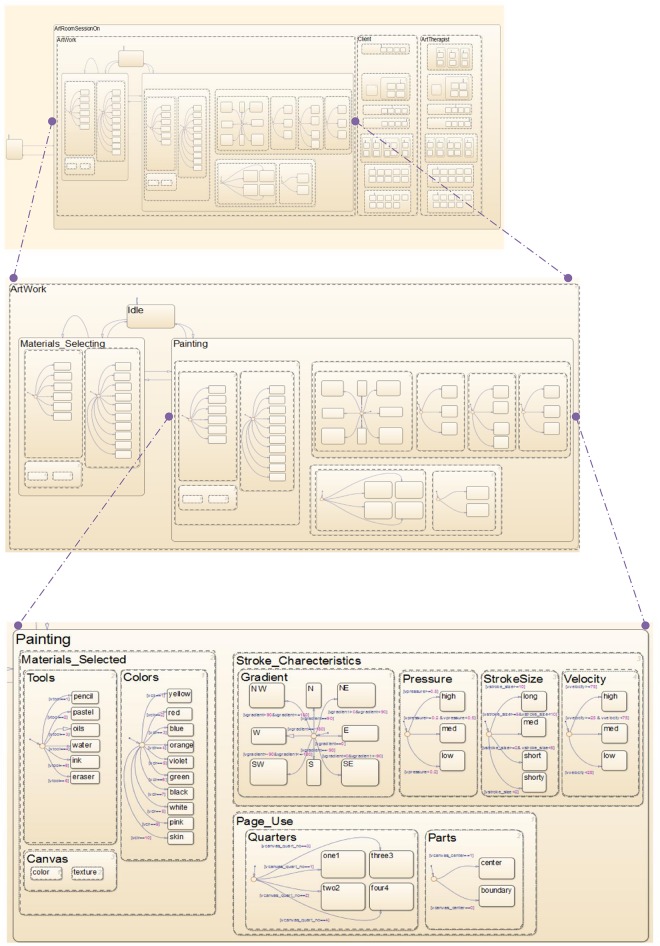
Hierarchical view of the system models. The visual modeling of the system using the Statecharts formalism. (***Top Panel***) The **Artwork** model is a state entity in the art room, concurrent with other states there; i.e., the **Client** (patient) and **ArtTherapist**. (***Middle Panel***) The Artwork’s creator can be in one of three states: **Painting**, **Materials_Selecting** or **Idle**. (***Bottom Panel***) The **Painting** state is further decomposed into sub-states formulating the art creation process.

We model the creation and interaction dynamics of the art room using Statecharts. The artwork, patient/client and therapist, the three main entities composing the art room, function and operate in parallel. Thus, as shown in Fig 2, they are depicted as concurrent states, **ArtWork**, **Client** and **ArtTherapist**, represented by dashed lines and encompassed by their parent super-state, **ArtRoomSessionOn**. The states **ArtRoomSessionOn** and **ArtRoomSessionOff** are represented by solid lines, and are mutually exclusive. The arrows represent the state transitions, and are labeled with events, which are recognized throughout the statechart model, and cause the transitions. Thus, the event **ESessionStatus** toggles between **ArtRoomSessionOn** and **ArtRoomSessionOff**. The top panel in Fig 3 shows the high-level states of the system. Zooming in shows the substates composing the **ArtWork** (middle and bottom panels) and the **Client** and **ArtTherapist** (S1 Fig).

The artwork is the center of creation in the art room and can exist without the need for a clinical setting. In the middle panel of Fig 3, the **ArtWork** state is decomposed into its substates, **Materials_Selecting**, **Painting** and **Idle**. The exclusive substates of **ArtWork** model the modes the creator can be in vis-à-vis his or her artwork. The creator enters the **Painting** state on the event of starting a stroke on the canvas. The state of **Painting** is left when the stroke ends; i.e., the drawing tool is lifted up from the canvas. At this point, the creator enters either the **Materials_Selecting** state, by selecting new tools or colors, or the **Idle** state. Exiting each of these states and entering the others is triggered by the successive events.

The internal dynamics while in the **Painting** state is rich, as seen in the bottom panel of Fig 3. Here we use the orthogonal/concurrent states **Materials_Selected**, **Stroke_Characteristics** and **Page_Use**. All three are active simultaneously when the artwork is being created, accounting for concurrent occurrences that are rather difficult to follow with the human eye, but are made plausible by the orthogonality construct of Statecharts within the tracking model.

Within these states there is further decomposition into substates. The **Materials_Selected** state, for example, is modeled by a range of color and tool choices that specify the current stroke being executed. The attributes specifying the canvas color and texture occur in parallel, and here their options are fixed. The stroke is also tracked for its drawing direction (gradient), the pressure exerted by the tool drawing it, and its size and velocity. These attributes are modeled as concurrent/simultaneous states within their **Stroke_Characteristics** parent state, and each of these is further described by exclusive substates. For example, considering the **Velocity** state, the velocity of the drawn stroke can be high, medium or low, depending on conditioned threshold values (appearing along the transition arrows). The **Gradient** is modeled in a compass-like manner, determined by the angle of the stroke. It is also important to track and analyze the **Page_use** in terms of the locations of the stroke. The page divisions of interest are the four quarters division (top left, bottom left, top right and bottom right) and the two parts division (center and boundary). A stroke is thus in states **Quarters** and **Parts** in parallel, and within these it is characterized by the exclusive substates.

In S2 Fig we provide a glimpse into the first stages in a possible model for a music session [55], showing the three concurrent states of **Music_Work**, Client and **Music_Therapist**, encompassed by their parent super-state, **MusicRoom_SessionOn**. The **Music_Work** state is decomposed into its exclusive substates **Idle**, **Selecting**, and **Playing**, with the latter state to further include the rich dynamics therein.

By employing Statecharts, the system’s possible behavior and those facets thereof that are of interest to the modeler or analyzer, are characterized in a clean and well-structured visual fashion, and are quite self-explanatory. Moreover, since the language has a formal syntax and semantics, both textual and graphical terms have precise dynamic meaning, so that the model can be analyzed for dynamic properties and simulated directly, or translated into fully executable code. As the overall working environment of our model we chose the MathWorks suite of Stateflow/Simulink/MATLAB [56–58], though one can also use Rhapsody [59]. Such tools enable the creation and execution/simulation of the model and provide analysis options that facilitate investigating the dynamic behavior of our system.

The ability to track, analyze and document the dynamics of the creation processes is reported in the context of our proof-of-principle artwork study described next.

## Proof-of-Principle Artwork Study

### Concepts and Use of the Computational Paradigm in the Artwork Study

**Fig 4:**
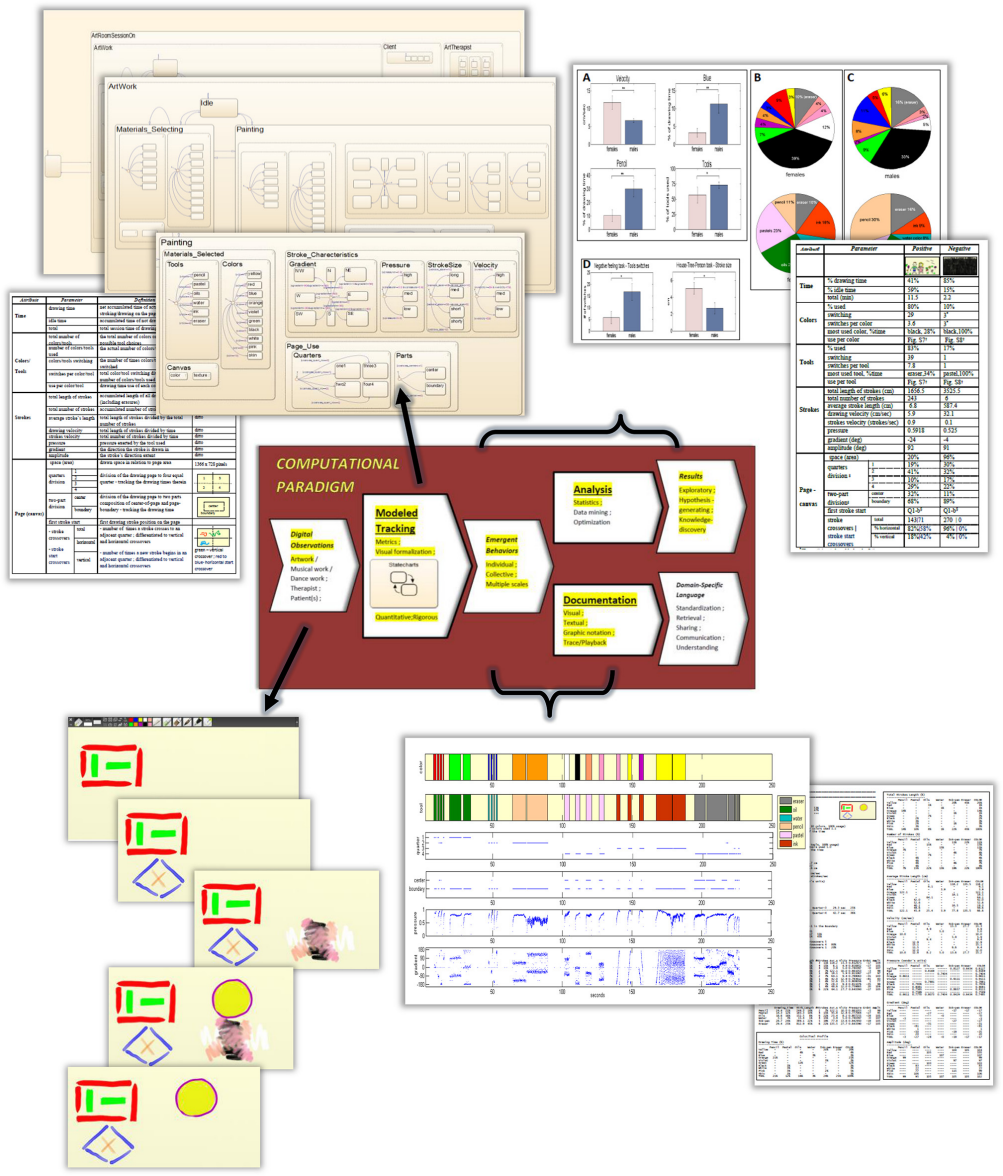
The paradigm applied to artwork. An illustration of the technology used in artwork: Tracking, analyzing and documenting dynamic processes in a human subjects study. Marked in yellow are the methodology’s capabilities utilized in this work.

Here we describe the CP and its applicability to the artwork study, showing a scheme of this use in Fig 4. In its center, the figure marks in yellow those capabilities of the method that are used and reported upon here (the unmarked ones are described in the Discussion section).

The art medium used is a computer tablet running a commercial vendor’s digital art software. The drawing is done directly on the tablet’s touchscreen by using a stylus pen that emulates our art materials: drawing tools and palette of colors. This apparatus aligns with the growing trend of using technologically in the arts and in therapy [60], and was chosen also for ease of mobility and sterility (such as in hospitals). The digital medium allows for diverse color and tool options and no depreciation and exhaustion of materials. For further details, see the Setup subsection in next section, ‘The Artwork Study Setting’. The bottom left-hand side of Fig 4 shows the artwork creation process as chronological snapshots of drawing on the tablet screen.

#### Modeled Tracking Module

**Fig 5:**
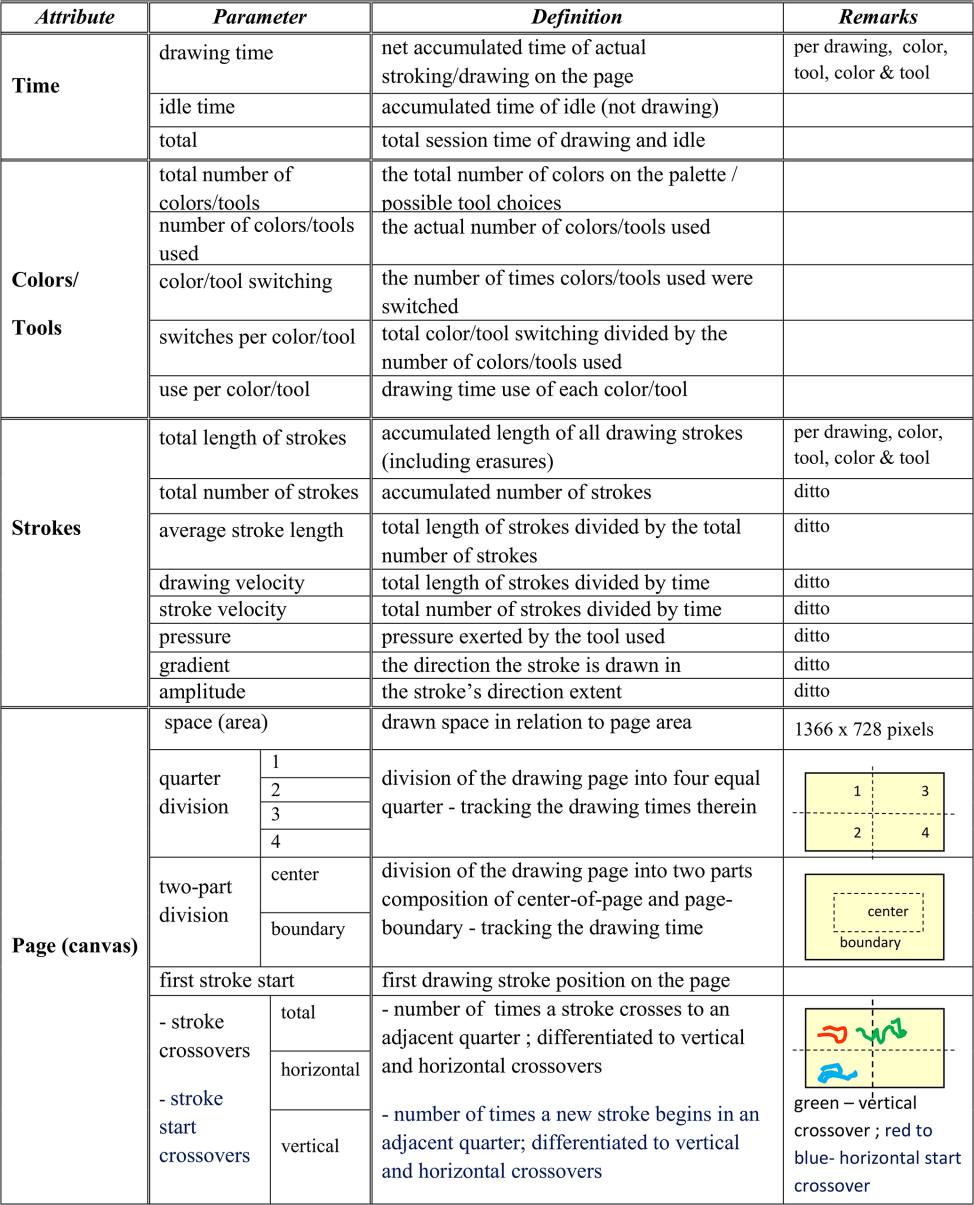
The table defining the parameters/metrics of the Artwork's construction process.

The inputs to our system, that is the digital observations in Fig 4, include the color and tool choices over time, the X and Y coordinates of the stroke being formed on the touchscreen (canvas), and the pressure exerted on the drawing tool. Within the Modeled Tracking module, these five basic parameters are used to compute additional parameters/metrics that quantitatively describe the dynamics of the creation process, its states and durations therein. Examples include the percentage of time a specific color or tool is used, the direction of the stroke and the page areas it is being drawn on. The metrics are shown in the table of Fig 5, and accompany the Statecharts model (top left-hand side of Fig 4).

#### Documentation Module

**Fig 6:**
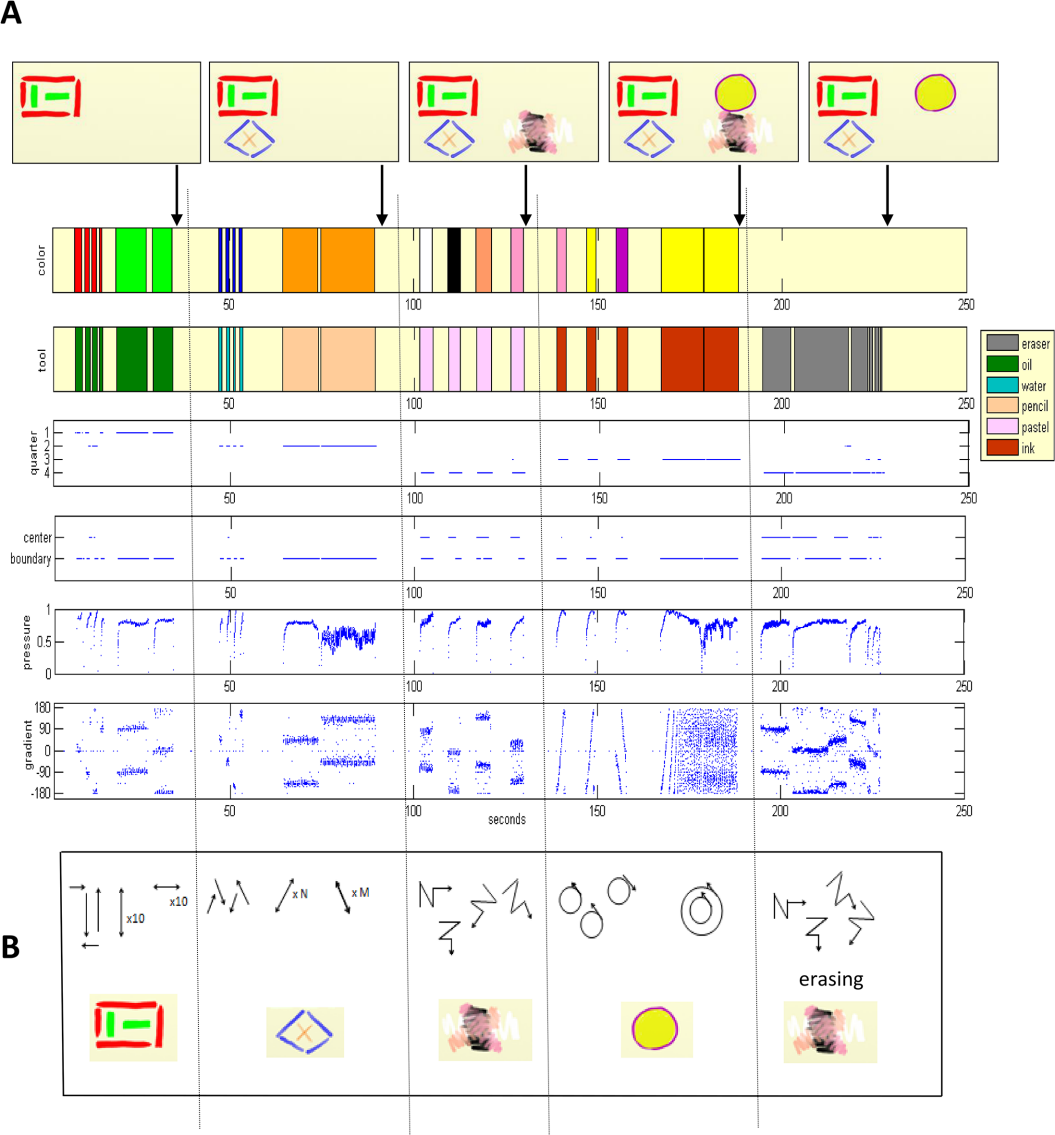
Visualization of the artwork construction dynamics. (**A**) The visual report of the dynamics of an artwork progression, displaying rigorous tracking of events and states of the artwork over time. For example, stroke start and stroke end, the color and tool used for the stroke, its location in the page (in which quarter and whether in the boundary or center), the drawing pressure and direction. Idle periods are clearly seen, as well as erasing epochs of drawn objects. Snapshots of the artwork creation appear along the parameters temporal plotting. (**B**) Illustration of the interpretation of the visual report, which enables the reconstruction and formation sequences of objects in the artwork.

**Fig 7:**
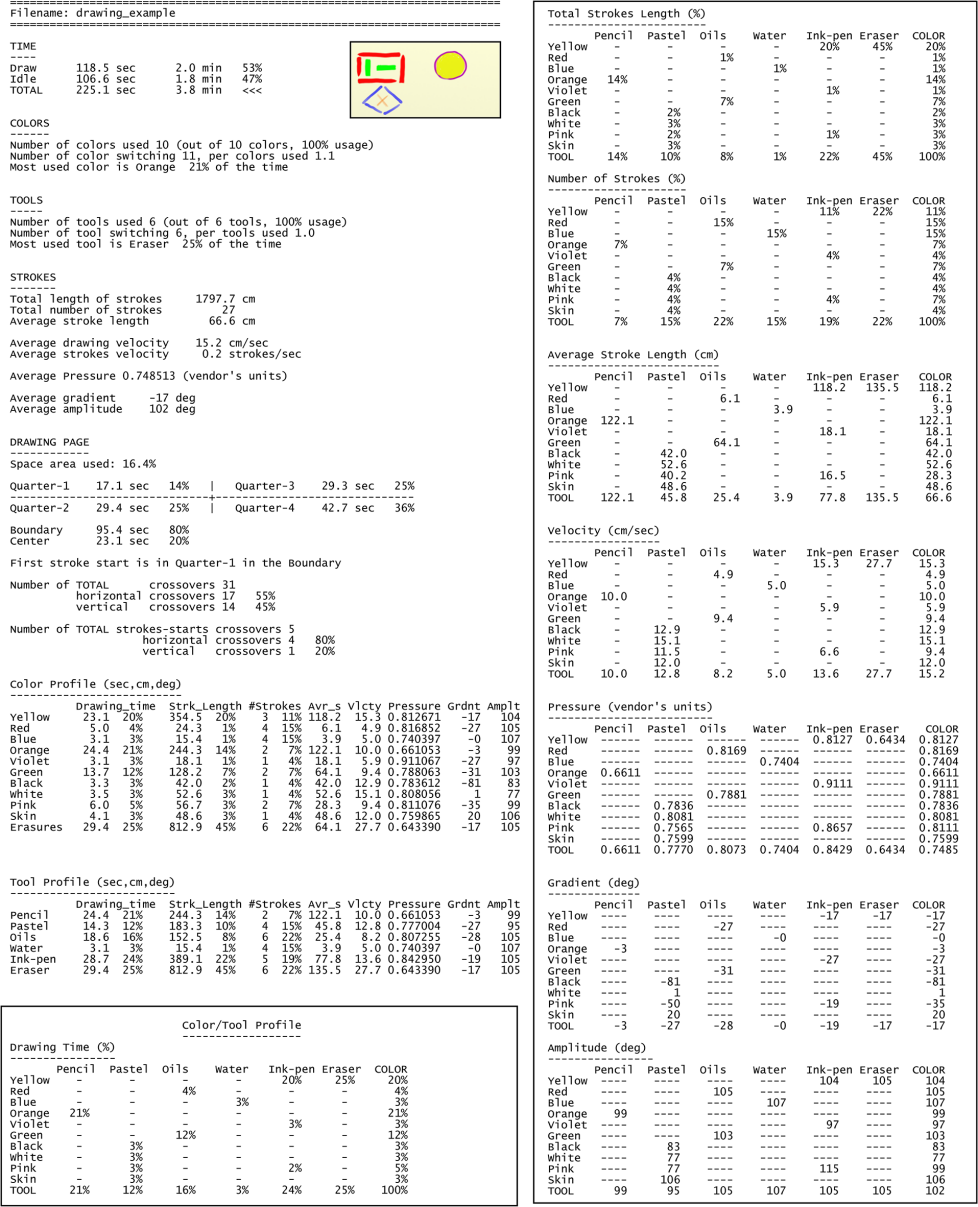
Quantification of the artwork construction dynamics. The textual report of an art creation process dynamics. It shows the quantitative tracking and analysis of an artwork’s creation specified by the parameters' table of Fig 5 and their cross-section calculations (the respective visual report of the creation dynamics is shown in Fig 6).

The dynamics of the drawing session are visualized in a temporal plot documenting the values of key parameters forming the artwork. As exemplified in Fig 6A, these are: colors, tools, page use (quarters, center/boundary) and strokes characteristics (pressure, gradient). This description allows the dynamics of the art session to be visualized on a single page, and facilitates reconstructing the sequence and way the artwork’s objects were created (see Fig 6B). Accompanying this is a detailed textual report with the quantitative information of the dynamics; i.e., the decoding parameter values and their cross sections. See Fig 7 for the textual report of the example in Fig 6. The drawing session itself is recorded in full, and can be played back in order to reproduce the process that led to the end-product artwork.

The temporal visualization of the creation process via its plotting enables tracking the durations and the order in which the objects were created, whilst recording the color and tools used to create them, and the page areas they were placed in. The gradient plot enables tracking the direction of the hand movement creating the objects and the pressure exerted in doing so. In the example of Fig 6A, the end product (rightmost drawing) has three major objects, a rectangle, a diamond and a circle. However, the documentation reveals that a fourth object (the strange-looking “pink-black” one in quarter-4 of the middle snapshot) was created at session time 100–130 sec, and was erased around 60 seconds later (the gray epoch in time, ca. 190–230 sec). This is seen to have been done by six eraser strokes, with diverse lengths. Strokes generated by using the eraser are also considered here, since they reform objects and modify compositions, and are also a crucial aspect of the session's dynamics.

The visual documentation of the process also enables reconstructing the exact way objects were created. In Fig 6A, the rectangle, for example, can easily be seen to have been drawn using oil colors in quarter-1 of the page, and the red color appears to dominate the green. Nevertheless, the visual report reveals that the two green lines were drawn using back-and-forth strokes that took more time than the straightforward clockwise direction by which the red rectangle edges were created. This can be seen in the visual description of the object formation in Fig 6B. Similarly, in the diamond drawing in quarter-2, the blue water color diamond (created in a clockwise direction) dominates the diagonal orange pencil lines. However, the report reveals that the latter took the longest time to create of all the objects in the drawing. In addition, the -135 to 45 degree diagonal was created first. Numerous counter-clockwise circular strokes in yellow ink were used for a period of ~30 sec, in the process of drawing the yellow face circle in quarter-3. It was initially outlined by a counter-clockwise circular contour in pink and yellow, which was then overridden by a violet clockwise-drawn circle.

The object drawn in quarter-4 (and later erased) can be reproduced in full, including its components and order of creation: First was the white zigzag stroke going from left to right, then the black element drawn from top to bottom, then the skin-colored diagonal and then the pink diagonal going from center to boundary. All four lines were drawn with increasing pressure.

Precise temporal information about the parameter values appears in the accompanying textual report, shown in Fig 7. Time measurements show that the entire drawing session of Fig 6A took 3.8 min, 47% of which were idle time; that is, the cumulative time in which drawing tools were not stroking the page. Hence, the actual drawing time is 53% of the total session. In this example, all the tools and colors customized on our drawing apparatus were actually used. As can be seen also in the visual report, the textual report shows that the orange color enjoyed the most use—21% of the drawing time; i.e., 24.4 sec. The eraser was the most often used tool—25% of the drawing time (29.4 sec). Stroke measurements show that the entire drawing was carried out using 27 strokes, with a total length of almost 18 meters (1797.7 cm), with an average velocity of 15.2 cm/sec and average pressure of 0.749 (digital art vendor’s units). Drawing page measurements show that the area actually used was 16.4% of the canvas, where 36% of the drawing time was spent in quarter-4 (the drawing and then erasure of the “pink-black” object). The compositional objects in the artwork are confined to the page quarters division. This is also manifested in the value of a parameter we termed ‘stroke start crossovers’ (Fig 5), counting the number of times a new stroke begins in an adjacent quarter—which is 5. Additional parameter values and their mutual cross sections appear in the textual report for further analysis and study. The Documentation module transforms the dynamic process of art creation into visual and textual reports. This is depicted in the bottom right-hand side of Fig 4, where individual emergent behaviors are documented as such reports.

#### Analysis Module

Within this module, we carry out our investigational artwork study in which we are interested in exploring the artistic expressive behavior of the participants in response to drawing tasks, and quantitatively comparing the dynamics of their creation processes. In addition, we wish to study the collective behaviors of the participants segmented into gender and age groups, where here, the creation processes are investigated and analyzed for discovering significance in the dynamic attributes, i.e., of the states’ profile and metrics calculated (as in Figs 3 and 5). For example, the duration of time a color and/or a drawing tool is used; erasures periods; the velocity of the drawing strokes, the strokes accumulated length, page usage, etc. The upper right-hand side of Fig 4 depicts some results we obtain in the collective studies and an individual's expressive behaviors comparison. These are discussed in detail in the following sections.

### The Artwork Study Setting

#### Setup

The art media the participants used was a Lenovo X230 computer tablet, and they drew using a stylus pen on a customized screen for the study (see S3A Fig). The tablet ran the ArtRage4 digital art software [61], which also outputs script files recording the drawing sessions. These were subsequently “read into” our Statecharts and analyzed by our methodology. The participants chose from a palette of ten colors: yellow, red, blue, orange, violet, green, black, white, pink and skin. They had a choice of six drawing tools: pencil, pastel, oils, water color, ink pen and eraser. All colors can be chosen for all tools. The drawing page (canvas) has an off-white yellowish color, in order for the color white to be visible. S3B Fig depicts the diversity of chromaticity, materials generating texture and other real world feel effects, such as blending and mixtures.

#### Subjects

Twelve participants, of whom six were male and six were female, with an age mean of 38.8 (SEM = 3.3), median 35.5. The participants had no formal art training or professional painting experience. They all came from similar cultural and academic backgrounds.

#### Ethics Statement

The research protocol was reviewed and approved by the Weizmann Institute’s Bioethics and Embryonic Stem Cell research Oversight (ESCRO) Committee. All participants signed a written informed consent.

#### Procedure

The individual participant was seated comfortably in front of the tablet, which was placed on a table. He or she was alone in the room with the experimenter present. The experimenter was seated next to the participant, but not facing the artwork or the participant. The participant was asked by the experimenter to produce artworks for four drawing tasks, to be consecutively carried out one after the other. The drawing tasks were not limited in time. The first task was a warm-up (calibration). The individual was asked to choose a single color and draw a line with this color by each of the drawing tools. In the second and third tasks the subject was asked to express in drawing a positive and a negative feeling, respectively (phase A). For the fourth task the individual was asked to draw an image of a house-tree-person (HTP) (phase B). Phases A and B were carried out with free choice of colors and tools. The order of these phases was switched between the participants to avoid emotional fixation. For example, a negative emotion may condition one’s reaction and may have produced a bad mood when drawing a positive emotion.

Before the actual drawing tasks, the participant was acquainted with the art tablet media by being allowed to use it freely with no time limit. Preceding this, the research intentions and full procedure were explained (see full instructions in S4 Fig). At the end of the procedure, questions were answered.

#### Statistical Analysis

The main ***results*** shown and discussed here are statistically significant for both the parametric student *t-*test for means and the non-parametric Wilcoxon rank sum test for medians (unless reported otherwise). The latter is equivalent to Mann-Whiteny *U* test. Statistical analysis was performed using MATLAB’s Statistic Toolbox [62].

### The Artwork Study Results

**Fig 8:**
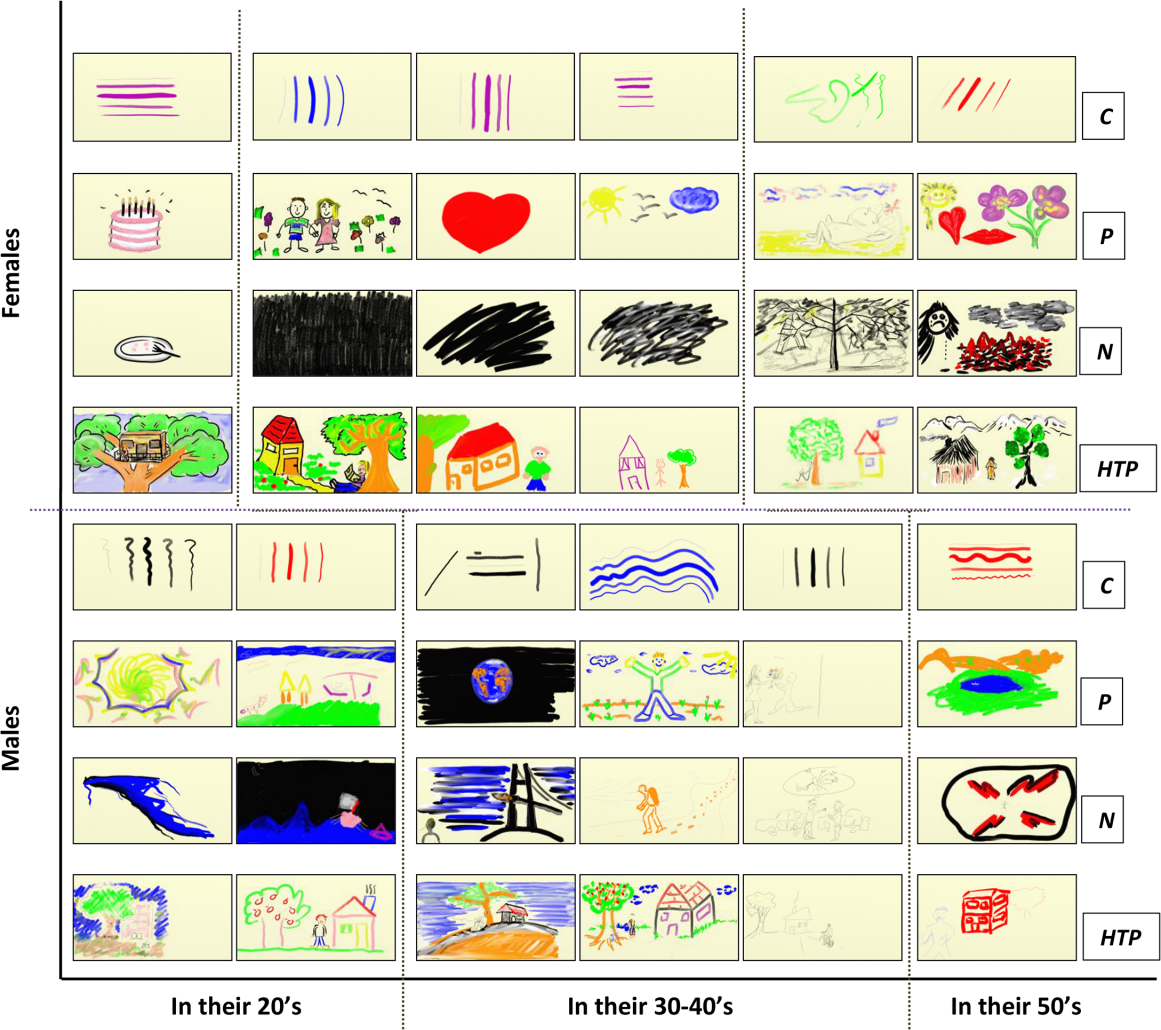
The participants’ artworks in the study. These are categorized according to the demographic attributes of gender and age. The ordinate is partitioned into males and females and the abscissa into age groups of participants in their 20s, in their 30-40s and in their 50s. The rows corresponding to (*C*) are the artworks of the warm-up calibration drawing task, (*P*) are the artworks of the positive feeling drawing task, (*N*) are those of the negative feeling drawing task, and (*HTP*) are those of the house-tree-person task. Each of the participant’s artwork are in his/her respective column.

The resulting artworks are presented in Fig 8, with each participant’s work being arranged in a column from top to bottom, categorized by gender, age and drawing task. An enlarged version can be found in S12–S15 Figs. Our analysis quantified the dynamics of the creation processes for individuals and collectives at multiple levels; i.e., in single and multiple drawing tasks, as described next.

#### Analysis of Individual Emergent Behaviors

**Fig 9:**
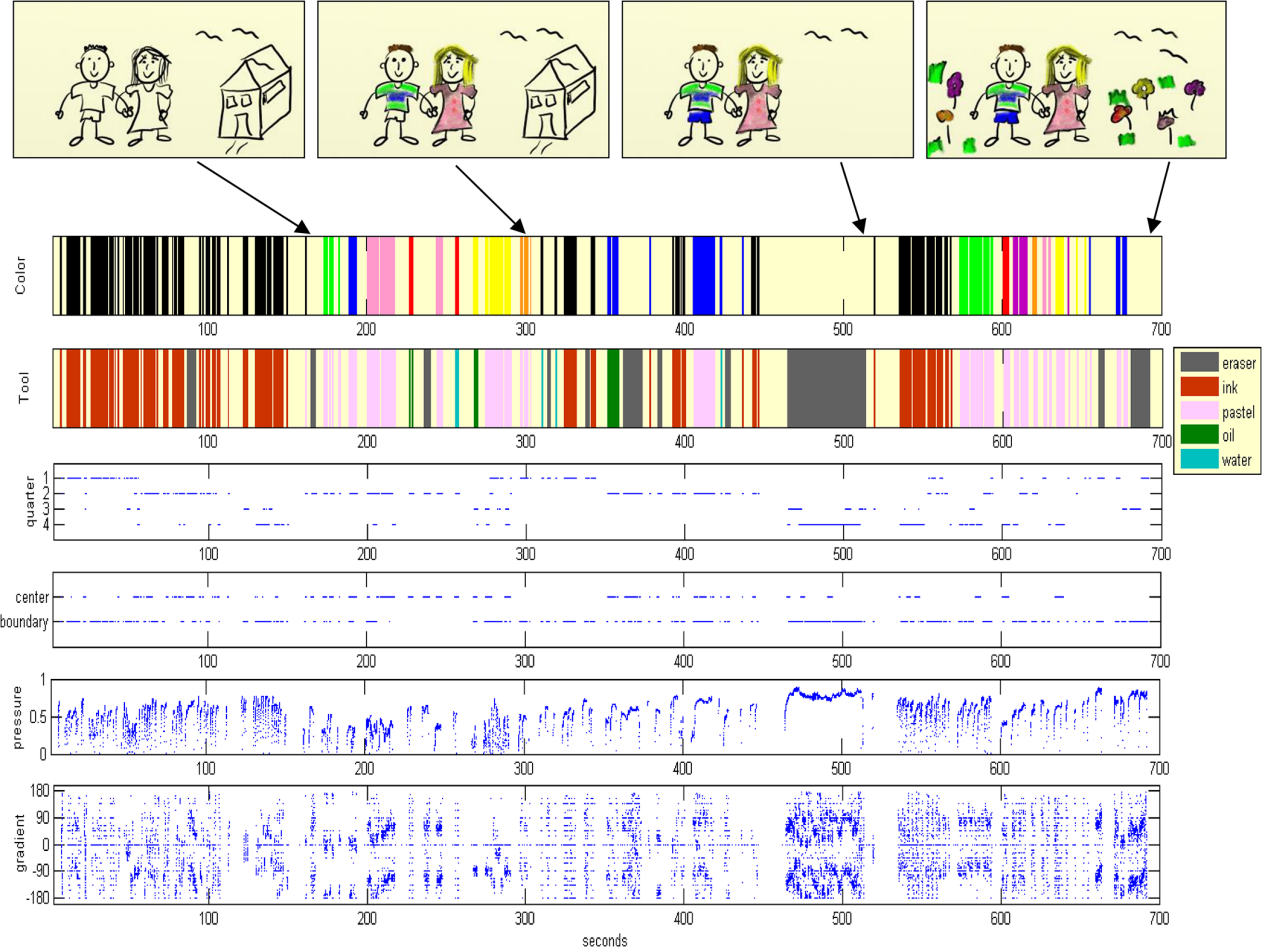
Visualization of an art making process (a positive feeling). A visual report of the creation process dynamics of an artwork imaging a positive feeling.

**Fig 10:**
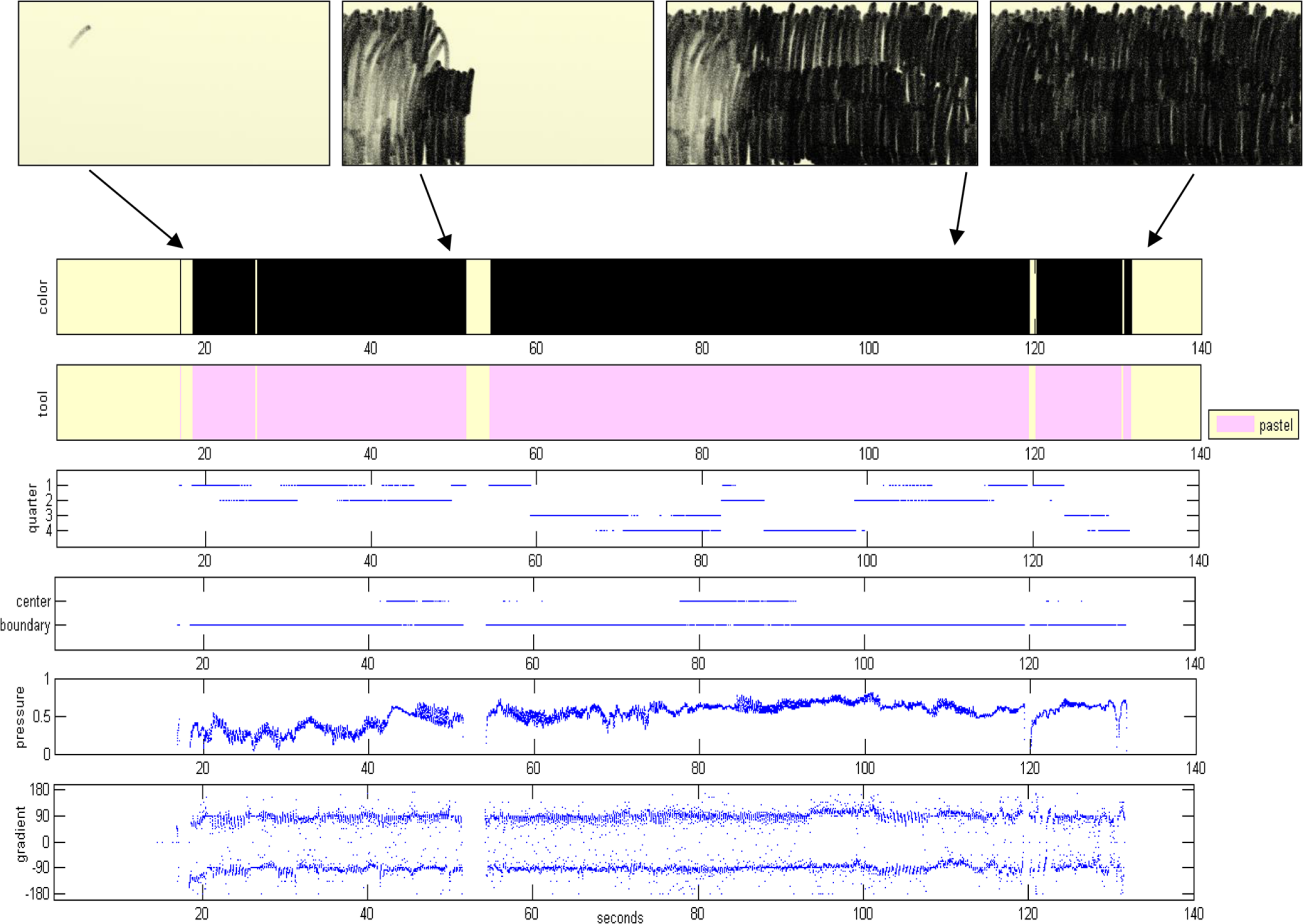
Visualization of an art making process (a negative feeling). A visual report of the creation process dynamics of an artwork imaging a negative feeling.

Here we present the enablement of our method to track, analyze and document the dynamics of art creations of individuals. We do so by showing and comparing the dynamics of the creation processes of an individual participant’s positive and negative feeling artworks. The creation process dynamics are “decoded” into the parameters/metrics appearing in Fig 5 and are presented in the visual and textual reports. The visual report of the positive feeling drawing of one of the participants (a female in her 30s) is shown in Fig 9 and the textual report in S5 Fig. The visual and textual reports of the participant’s negative feeling drawing are shown in Figs 10 and S6, respectively.

Tracking the dynamic processes of creation and erasure revealed that the end product of the positive feeling image of Fig 9 had changed during the course of its creation. Erasures took a notable portion of the drawing time, around 1/3 (S5 Fig), mostly due to a contiguous erasing epoch of ca. 45 seconds beginning around session time 465 seconds (Fig 9). These were carried out with the high pressure of 0.61 (above the average of 0.59 for this drawing), with fast hand movements and the high velocity of 8.6 cm/sec (well above the average of 6.8 cm/sec). The erasure part is 50% of the total stroke length of the drawing; i.e., 833 cm out of ca. 1660 cm for the entire process, done by 6% of the total number of strokes, that is, 14 long strokes out of 243 strokes needed to create the final drawing. Our tracking shows that the house drawn next to the human couple in Fig 9 was totally erased and a colorful field of flowers was drawn instead. In this drawing, 83% of the tools were used. The pencil was the only tool that was not used, and the tool used most was the eraser, taking up 34% of the drawing time. The pastels and ink pen were the next preferred ones, with 33% and 28% of the drawing time, respectively. Although the image drawn is highly colorful, using 80% of the available colors, black was the color most used, 28% of the drawing time (27% of it with the ink tool). The ink pen was used mainly for drawing the outlines of the illustration and the pastels for coloring within. Interesting also is the notable amount of drawing time spent (41%) in quarter-2 of the page. This amount of time was consumed by the drawing of the human couple and the coloring and tools investigations of their clothing.

Analysis of the image represented in the negative feeling drawing task of Fig 10 shows that the black "mass" was created by fierce and lengthy vertical movements (temporal gradient of ±90 degrees with a total average of -4 degrees), and with growing pressure. Most of the page was covered by four black strokes of pastel, with the drawing medium rarely lifted up from the page for the entire session (85% drawing time and only 15% idle time; S6 Fig). The strokes were generated locally and continuously, with no strokes beginning at adjacent quarters (stroke start crossovers is zero), but with 96% of the stroke crossovers being horizontal, and at the very high velocity of 32.1 cm/sec. The end result is a painting with total stroke length of more than 35 meters and average stroke length of more than 5 meters.

**Fig 11:**
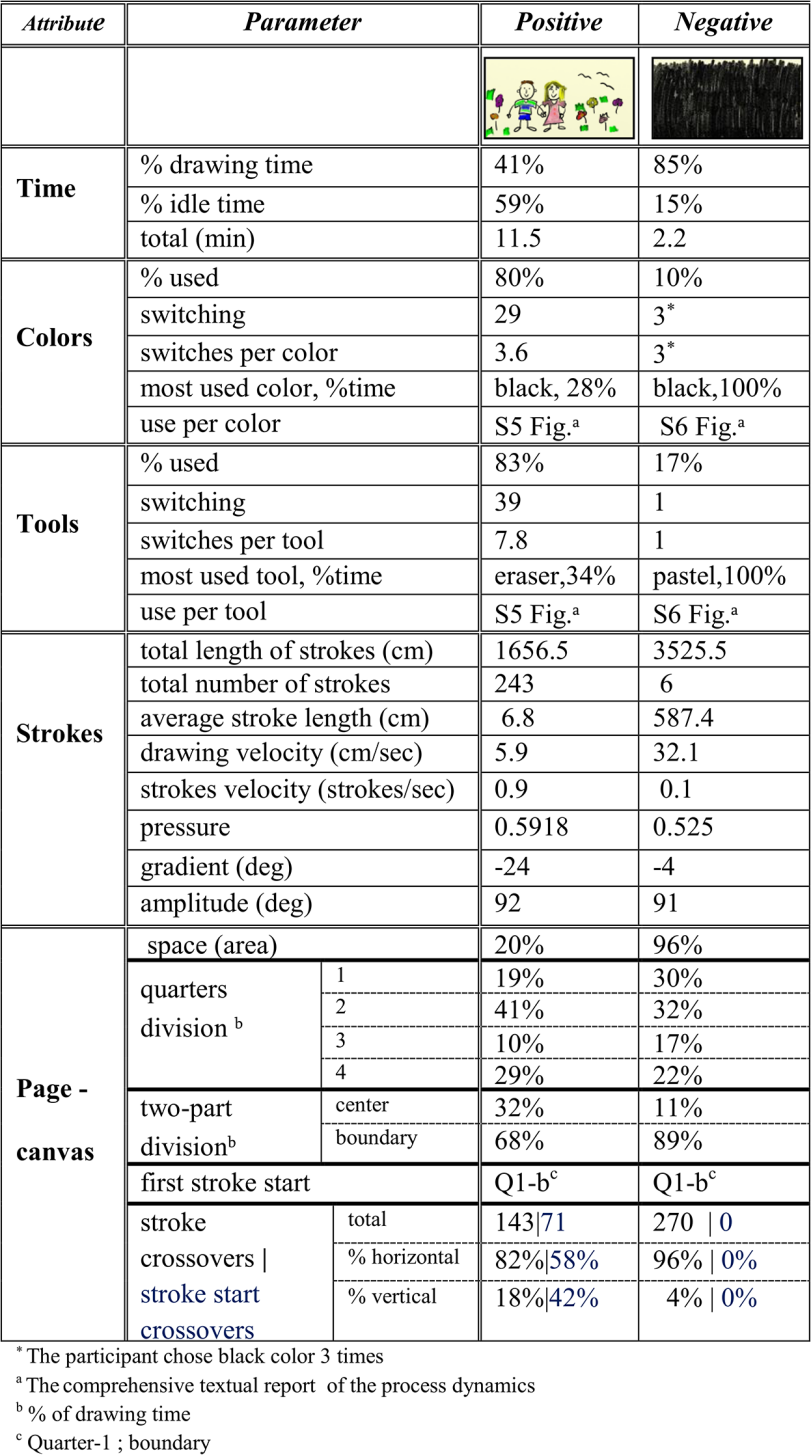
An example of parameters/metrics comparison of an individual's artworks construction processes. The processes compared are of the creation of a positive feeling image and that of a negative feeling.

One of the things made possible by our computational approach is to compare the creation processes of several artworks. The table of Fig 11 displays how this is achieved for the dynamics of the previous two drawings. The quantitative comparison therein shows clearly that whereas the positive feeling image is an expression of a narrative or symbolic elements, the negative feeling image is an abstract composed by contiguous movements in black. Out of the total session time of each artwork, the positive image actual drawing consumed 41% of the time compared with the 85% the drawing time of the negative image. The latter artwork covering 90% of the page in one fifth of the total session time (2.2 min), compared to the positive one (11.5 min), and being carried out with an average velocity that was 5-fold higher than that computed for the positive image. The negative image has a total stroke length twice longer than that of the positive image, but required only 2% of the number of strokes of the latter. In the next section we generalize this comparison to studying collective comparisons and behaviors.

As shown earlier, tracking the creation dynamics also allows us to discover important phenomena that are otherwise missed when examining only the end art product. For example, in one of the positive feeling drawings our computations show the use of white 65% of the drawing time, although this is not seen in the final drawing. Examining the reports of the creation of this particular artwork (S7 Fig) reveals that the participant had been blending in the white color in repetitive circular motions for most of the drawing time. According to projective art literature, repetitive behavior within art making is an expression of stress [63]. Another interesting example is of one of the house-tree-person artworks, where erasures consumed 43% of the drawing time. The reports (see S8 Fig) and playback reveal that the participant had modified the house twice and the person six times, changing the colors, tools, forms and dimensions of these images, until the final output was produced. This provides valuable information, e.g., for evaluation and diagnosis, as well as for understanding the overall dynamics of creating images [64]. It would most probably not have been properly noticed if the dynamic process was not tracked and documented.

#### Analysis of Collective Emergent Behaviors

**Fig 12:**
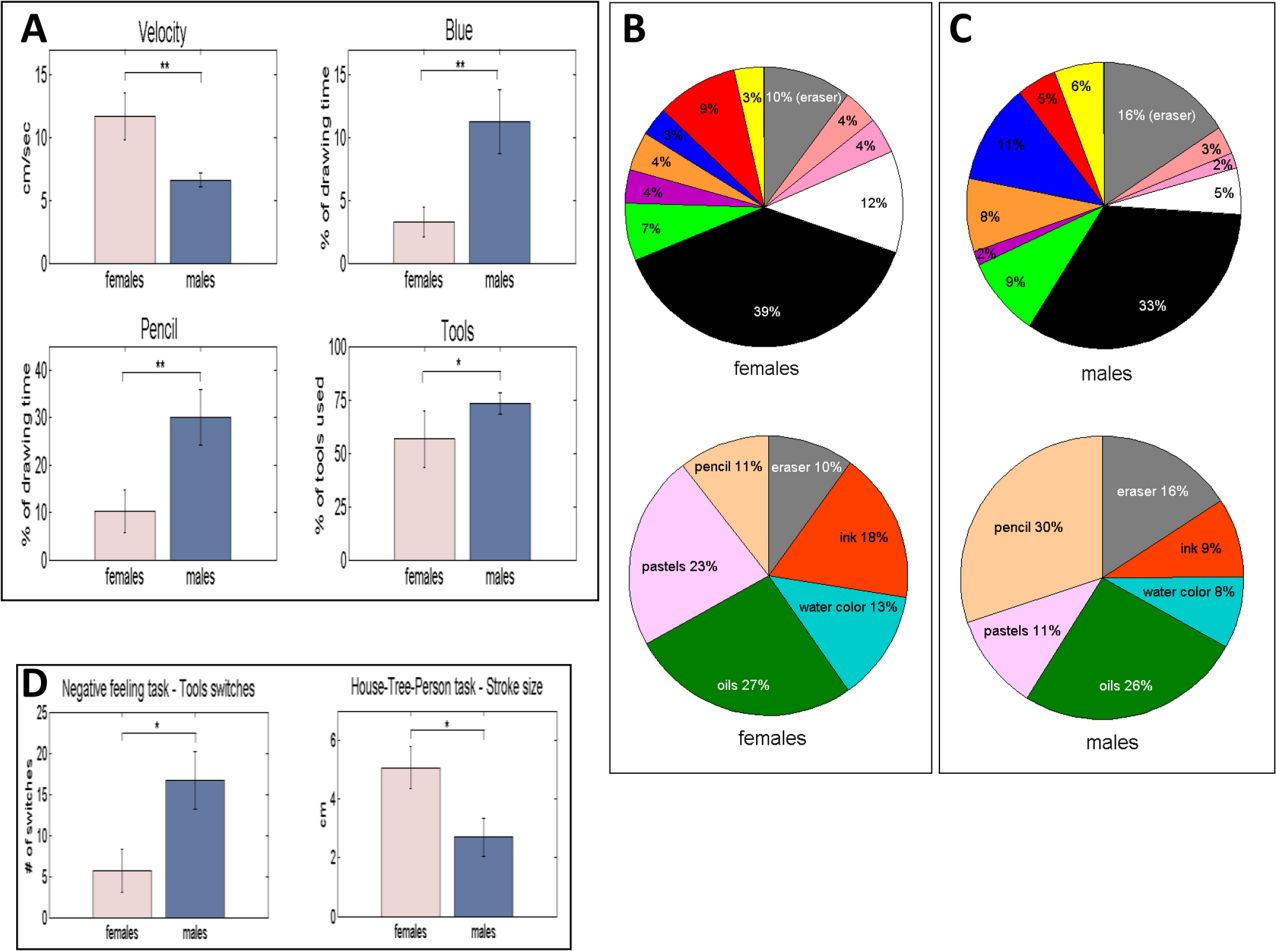
Depiction of gender difference. (**A**) Depiction of statistically significant mean differences in the gender study of the artworks. These have emerged for the mean drawing velocity, averaged over all artworks of males and females (*n* = 48), for mean percentage of time use of the blue color and of the pencil (*n* = 36), and for the mean number of tools used by both genders, depicted here as the percentage of total tools (*n* = 36). (**B**) The color and tool use of females, displayed as the percentage of drawing time. (**C**) The color and tool use of males. (**D**) Gender significance examples within specific drawing tasks. Mean difference in the number of tool switches has emerged for males and females imaging negative feeling. The artworks in the house-tree-person drawing task yielded mean differences in the average stroke size (*n* = 12). Data are reported as the mean ± SEM. *p < 0.05, **p < 0.01.

*Gender Difference Investigation*: The drawing tasks’ artworks were categorized by the creator’s gender. We investigated the differences between females and males in the means and medians of the parameters of Fig 5. The significant results are shown in Fig 12A **.** The top left panel shows that the drawing velocity of females was almost twice as high as that of males: 11.7 cm/sec vs. 6.6 cm/sec (*p*-value of 0.0084). We also found that the velocity averaged for females was higher than that of males in each of the four drawing tasks. In addition, females exhibited larger drawing times, computed as the percentage of the total session time. It is 42%, compared to 33% for males, in all three major drawing tasks combined (positive feeling, negative feeling and HTP), and was also higher in each of the tasks alone. As shown in the top right panel of Fig 12A, the usage time of the color blue computed as percentage of drawing time, was around 3-fold for males over females; that is, 11.3% of the drawing time for males vs. 3.3% for females (*p* = 0.0042). The blue color usage was higher for males also in each of these tasks separately, and the same goes for yellow usage. See S9A Fig, which also shows that the mean time usage of the white color for females was higher than for males in all tasks.

As to the use of drawing tools, the bottom left panel of Fig 12A shows that the pencil was used by the males three times more than by the females (30.1% and 10.3%, respectively, *p* = 0.0056). S9B Fig shows that males erased more than females and that females used water colors more than males. The percentage of drawing tools (from all the available ones) used by males was 73%, which is significantly higher than the 57% for females, with *p* = 0.0188 (Fig 12A bottom right panel and S9C Fig). The respective median differences corresponding to the mean-significant differences are also statistically significant.

The average use of colors and tools for females and males is presented in Fig 12B and 12C, respectively. It can be seen that the most-often used colors for both genders, computed as percentage of drawing time, was black: 39% for females and 33% for males. As reported, males erased more than females, and used erasures as the second most used “color”, for 16% of the drawing time. The third and fourth most used colors for males were blue and green: 11% and 9%, respectively. For females, the second most used color was white: 12% of the drawing time. Erasures (10%) and red (9%) were their third and fourth most used colors.

In terms of tool use as percentage of drawing time, both genders preferred the oils as the most used tool of choice: 27% for females and 26% for males. Females continued to prefer a “coarse” medium tool, using the pastels for 23% of the drawing time, whilst males used a more “controllable” medium as their second most used tool, the pencil, for 26% of the drawing time. For females, the pencil was the third most used tool (11%) and for the males it was the eraser (16%).

**Fig 13:**
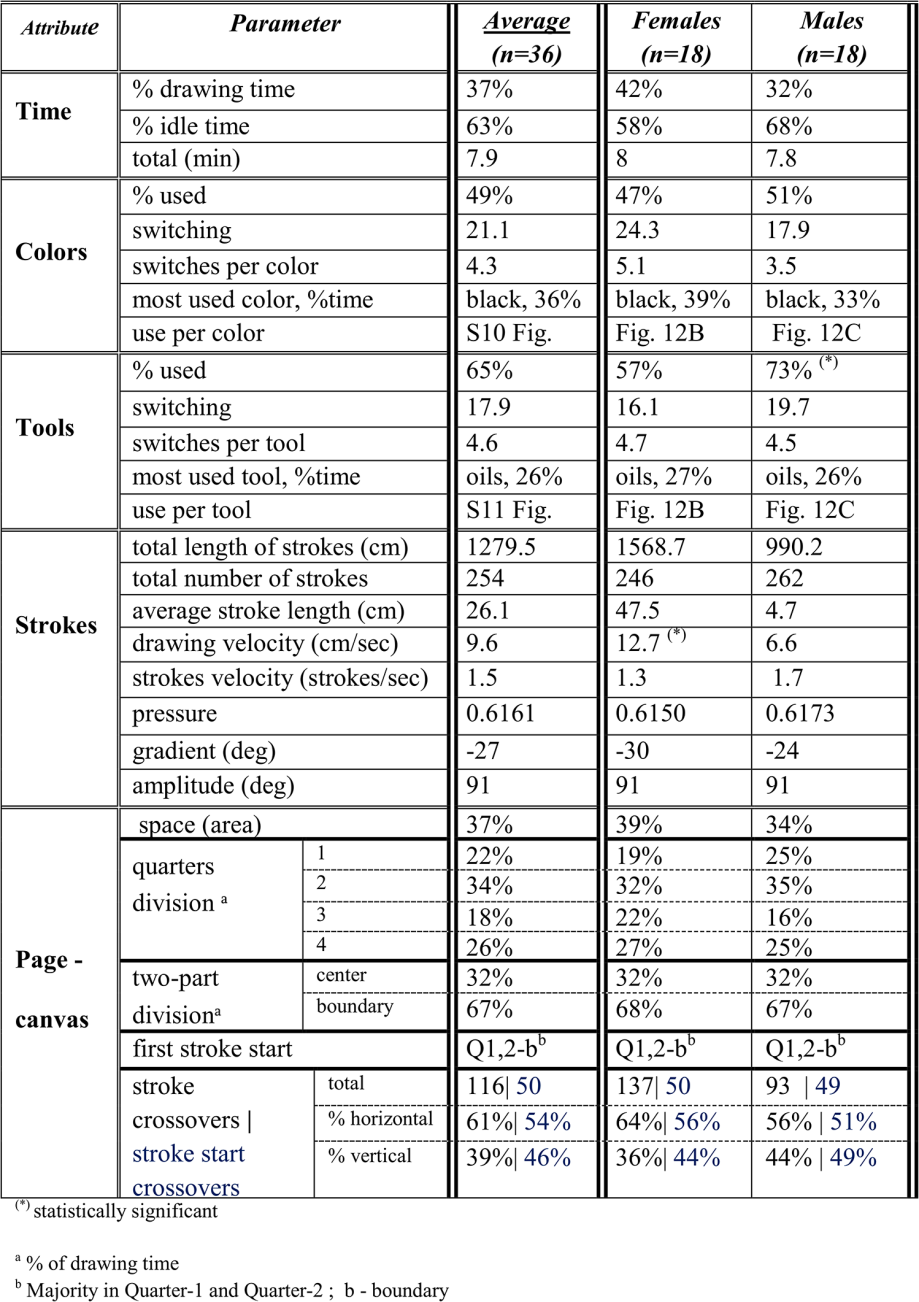
An example of artworks’ parameters/metrics construction processes comparison of female and male collectives.

Additional differences can be found in the table of Fig 13. Although females used fewer colors than males (47% vs. 51%, respectively), they switched between them more often than the males: 5.1 switches per color for females and 3.5 for males. Females drew for ca. 1569 cm in total length of strokes, in comparison to the males' 990 cm, and using fewer strokes (246 vs. 262, respectively). Compared to males, females also exerted less pressure whilst drawing, with a 6 degree bottom right shift in the average drawing direction, and used 5% more page space area. Notably, is the session’s drawing time percentage spent in the upper left quarter-1 for females (19%), which is the less preferable one of the four quarters. The less favoring one for males is the upper right quarter-3 (16%). Females having 137 horizontal and vertical strokes crossovers compared to the 93 of males; that is, 47% more strokes crossing to adjacent quarters. With respect to the time utilization of page area, both genders showed similar usage of the center and boundary portions of the canvas.

*Calibration Drawing Task* (S12 Fig)—Overall, females chose to use 40% of the colors and males 30%. Females did not choose black whereas males did not choose violet. Only blue and red were chosen by both genders.

*Positive Feeling Drawing Task* (S13 Fig)—The mean percentage of the tools used in this task is significantly higher for males: 86.7%, compared to 63.3% for females, with *p* = 0.0493 (S9C Fig). An additional interesting result is that of the males using the first page quarter (upper left) for 28.7% of the drawing time, which is significantly higher than the 20.0% for the females (*p* = 0.0503). Males used the colors orange and green for significantly longer times than females. Orange was used by males around 17-fold more than by females: 8.7% vs. 0.5% of the drawing time (*p* = 0.0340). For green the numbers are 17.3% for males, which is almost 12-fold higher than the 1.5% for the females, with *p* = 0.0260 (see S9A Fig).

*Negative Feeling Drawing Task* (S14 Fig)—As seen in left panel of Fig 12D, the males switched between tools 3 times more than the females: 16.7 tool switching for males and 5.7 for females (*p* = 0.0157). This is consistent with the observation that females were busier drawing, since their velocity is almost 4-fold higher than males: 22.2 cm/sec vs. 6.3 cm/sec.

*House-Tree-Person Drawing Task*. ***(*** S15 Fig)—In this study the average stroke size was found to be gender significant (right panel of Fig 12D), with that of females being almost twice longer than males: 5.1 cm for the females vs. 2.7 cm for the males (*p* = 0.0171). Males used the pencil with total drawing time almost 6-fold more than females: 40.3% vs. 7%, with *p* = 0.0114 (S9B Fig). The colors orange and green were found to have gender significance here. Females used orange for 13% of their drawing time whereas males used it around of a third of that; i.e., 4.2% (*p* = 0.0289). Green was used by the females more than twice as much as by the males: 22% of the drawing time vs. 9.7% for the males (*p* = 0.0195). See S9A Fig. In this task, orange and green were found to be significant for the females, whereas in the positive feeling task these colors were significant for the males. This may indicate that color choices are more determined by the drawing theme than by personal preference. An additional finding is that the females chose to place their first stroke in the center of page far more than the males did: 66.7% vs. 16.7%, with *p* = 0.0470 (no significant median difference for the latter).

**Fig 14:**
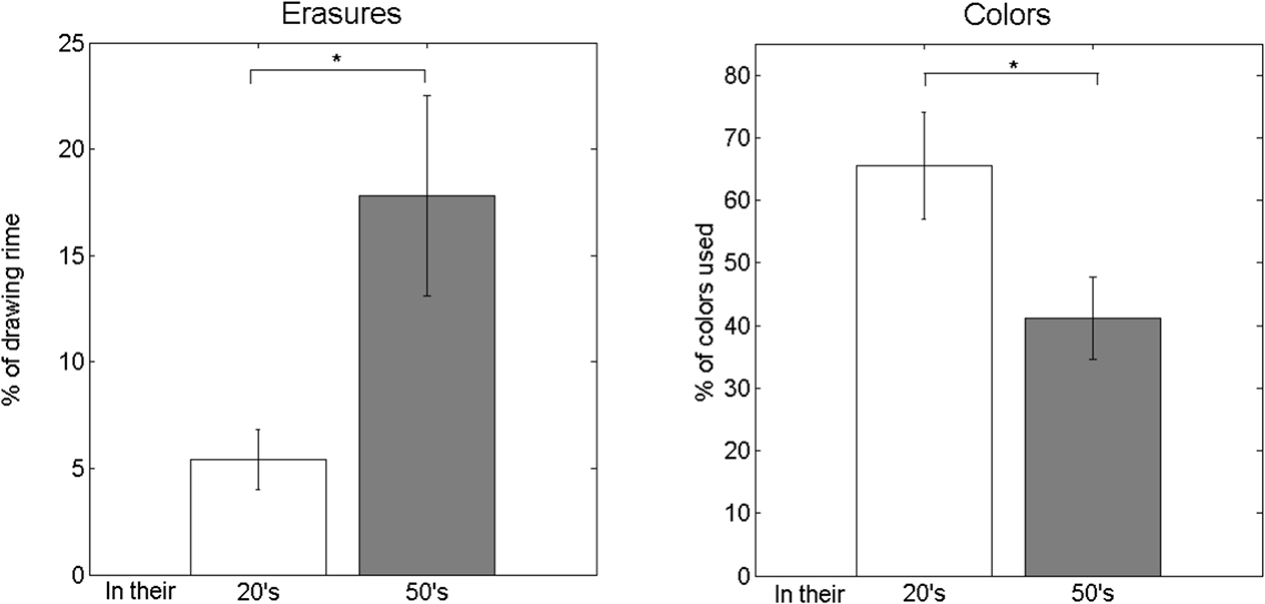
Depiction of age-significant differences. Differences in the means of parameters have emerged for participants in their 20s and in 50s. Mean differences are shown in the percentage of use time of the eraser (*n* = 18), and in the number of colors used, computed here as the percentage of colors used (*n* = 18). Data are reported as the mean ± SEM. *p < 0.05.

*Age Difference Investigation*: We analyzed the data to seek age differences of significance, within two clearly distinguishable groups of ages: participants in their 20s and participants in their 50s (see Fig 8 for the grouping). Two parameters out of the fifty we explored were found to have significant mean differences between these two age groups, as shown in Fig 14. First, the older group of subjects, i.e., those in their 50s, carried out erasing 17.8% of the drawing time, which is almost 3-fold the erasure time for the younger group, which was 5.4% (*p* = 0.0159). Second, the younger age group used almost 60% more colors than the older age group: 65.5% vs. 41.1% of the color palette, with *p* = 0.019.

*Collective Preferences Investigation*: By averaging the color and tool choices of the participants over the three major drawing tasks (positive, negative feeling and HTP), their collective preference emerged. This is seen in S10 and S11 Figs for color and tool use, respectively. The most used color is black, which was used 36% of the drawing time. This is mainly due to using black in the negative feeling drawing task, but not only that: it is also used for drawing sketches, contours, outlining and painting. It turns out that erasures were the second most used “color”: 13% of the drawing time. Surprising also is the use of white and green, which ranked as the third and fourth most used colors: 9% and 8%, respectively. The green and skin colors were not used by any of the participants in the negative feeling drawing task. The most preferred drawing tool was that of oil colors: 26% of the drawing time. Next was the pencil with 20%, and then the pastels with 17%. Whereas the pencil is considered a “tool of control”, oils and pastels are much coarser and less accurate, allowing the strokes to be made more freely and expressively. This is seen clearly in the negative feeling drawing task, with its high use of oils

In addition, as seen in the table of Fig 13, each participant’s drawing session was on average 7.9 min in duration, 63% of it being idle. While actually drawing, the velocity was 9.6 cm/sec, with 1.5 strokes made per second. The preferred page quarter was the second one (bottom left), taking up 34% of the drawing time. Fifty stroke start crossovers were carried out, around half of which were horizontal.

## Discussion

### Summary

In this work we introduced and illustrated the use of a broad research method based on computerized modeling, which allows for rigorous and quantitative tracking of behavioral dynamics generated in the use of expressive arts, and their objective analysis and documentation.

Our paradigm, the modeling of which centers on the visual state-event language of Statecharts, consists of three processing modules and the information that flows between them that originates from non-human digital observations. These are decoded into emergent behaviors via the Modeled-Tracking module, which hosts a Statecharts-based model of the explored system, to be subsequently analyzed and documented via the respective Analysis and Documentation modules (see Fig 1). Our empirical infrastructure enables one to conduct exploratory, knowledge discovery and hypotheses generating studies. Furthermore, the method's capabilities make it possible to investigate emergent behaviors of individual creators/patients and collectives of segmented populations.

We were able to illustrate the use and strength of the method by applying it to a proof-of-principle study of artwork investigations with human participants. We explored individual and collective emergent behaviors in response to several drawing tasks, by capturing, analyzing and documenting these (see Fig 4 for a summary scheme).

The Modeled-Tracking module captures and decodes emergent behaviors and includes: *(i)* measuring exact time durations of occurrences within the art session; e.g., net idle time in which the creator/patient is not engaged in art activity, and net drawing time, in which she/he is; *(ii)* tracking erasure periods and their time durations, as well as the usage of other drawing tools and color choices, their preference profile and cross-sections therein; e.g., colors palette per drawing tool, and also capturing and analyzing the switching frequency of colors and tools; *(iii)* capturing the characteristics of hand movements (strokes); e.g., jumpy, sharp, smooth, repetitive, etc., and calculating the drawing velocity, direction, amplitude, and pattern; e.g., circular—clockwise or counter clockwise, as well as the number of strokes generated, their total accumulated length, average size, and pressure exerted on the drawing tool and the erasure; *(iv)* tracking the drawing page (canvas) usage in time and space area, analyzing the art making process; e.g., whether it is carried out in a confined area (say, page corner or section), on the page boundaries or in its center, and also whether the drawing process involved crossing the page more horizontally or more vertically.

Furthermore, we were able to obtain gender- and age-significant results, using the Analysis module to explore collective behaviors for demographic differences. These include the findings that the drawing velocity of females was almost twice that of males, whereas males used the color blue and the pencil tool around three times more than females, and overall used more drawing tools. The significant results related to age include the findings that the participants in their 50s erased almost three times more than the ones in their 20s, whereas the latter used more colors. These gender- and age-significant hypotheses may point to factors that account for expressive variation impacted by art use, which can be further explored on larger scales.

The accompanying reports of the art drawing sessions output from the Documentation module exhibit uniformity and standardization, which enable the exploration and the comparison of the different artwork construction dynamics.

We thus provide comprehensive, quantitative and non-verbal analysis of art making. This rigorous capturing and analysis of the creation dynamics is important in evaluation and diagnosis, for example, where events and time durations are likely to be missed if one relies on the human observer, or when focusing only on the end product or final imagery. Hence, the meaning of art manipulation can be more carefully unraveled and can be based on empirical findings, as we have shown here.

### Future Goals

We aim to further solidify the methodology, tailoring it to systematic and mechanistic behavioral investigations, and to provide broad empirical evidence of the uptake of art-based therapies and their exerted effects. We shall also gear our research toward ameliorating and optimizing the use of the arts in the clinical setting. We believe these will be achieved by expanding our computational approach to allow additional real-world studies, and by the development of a domain-specific language. These issues include addressing the unmarked capabilities in the center of Fig 4 as discussed next.

#### Modeled Tracking Module

In addition to the artwork model, our Statecharts-based computational approach is to account for modeling the musical work, dance work, and their use in clinical settings involving the therapist and patient. A glimpse of the preliminary development of some of these models can be seen in S1 and S2 Figs. The use of Statecharts serves as a formalism for both modeling the system and designing a test bed for behavior capture and performance analysis. Moreover, we believe that in the future the Statecharts formalism will also become useful as the underlying blueprint of a training simulator for therapists, where the therapist plays the role of the environment, by responding to events as the model’s execution progresses.

The digital means responsible for the non-human observations are the input data to the system, which feed the tracking models. Whereas the tracking model and events and states are generic, the digital means we choose to feed the system with can be substituted, depending on particular development aims and studies. As described earlier for the artwork model, we used a computer tablet screen as the participant data input to the system. We may also use video recorders and other dynamic sensors to observe the artwork, and to capture the occurrences and usage of real art materials. In music and therapy, there is also a growing use of tablet screens. Hence, we plan to first input the musical work model with data collected from a tablet running digital music software. Our non-human observation of the input data may evolve to digital audio via sensors and microphones, which will identify and observe the use of real musical instruments.

Our computational paradigm will be extended to capture events and states also in 3-dimensional space, especially those relating to creation processes; e.g., sculpting, and those of bodily and auditory dynamics, narrating social interaction; e.g., facial expressions. Thus, body postures, body language, positions in the clinic room, etc., will all be part of the 3D movements in space tracked via the patient and therapist models (see S1 Fig). In addition, dance movements will be tracked by a future dance work model. For these tasks, movements need to be digitally observed and decoded. We will do this by videoing the clinical setting to derive the relevant 3D data. By employing state-of-the-art computer vision methodologies [65–73], we believe that the identification of movement types can be automatically achieved. For example, we could adopt algorithms for action recognition, facial expression identification, and hand positioning. For feeding the system with auditory occurrences; e.g., crying, laughing, yelling and singing, audio identification techniques will be used, such as sound recognition and analysis [74]. Our movement formalization work will be facilitated by employing the notion of *movement notation* [75–77]. The movement capturing and analyzing challenges will be met by adapting computer vision and robotics methods used in biomolecular medicine for solving 3D articulated object recognition problems [78,79].

#### Analysis Module

The Analysis module is study-based. That is, the emergent behaviors stemming from the Modeled Tracking module are analyzed per study. As seen in Figs 1 and 4, and in the proof-of-principle study, this is done using mathematical, computational and algorithmic means; e.g., statistics [62], data mining and optimization, depending on the study at hand. We wish to apply the approach throughout the technology’s development in a variety of arts-based studies with human subjects, ranging from focusing on the artistic construction work to investigations that examine clinical sessions and treatment improvement. These will include:

1. Studies that concentrate on the process of the construction itself and its measurements, and which also examine healthy-participant/patient related factors (e.g., age, gender, ethno-cultural, illness) that account for variation in responsiveness to arts intervention. These will utilize the artwork, musical work and dance work models; The demographic investigation reported here also validates the artwork model for additional studies and is an initial step in a broad investigation for discovering demographic differences in the dynamics of image creation when using diverse arts modalities. Hence, subsequent to developing the models for musical work and dance work, we will also explore demographic differences in the dynamics of auditory and movement image creation. An interesting phenomenon to investigate is that of synesthesia, a neurological phenomenon, in which stimulation of one sensory/cognitive modality pathway leads to automatic and involuntary experiences in a second one. Thus, a comparative study between the arts modalities is planned also for discovering synesthetic connections between attributes representing the demographic differences. Furthermore, comparison between the dynamics of image creations representing emotions and counter-emotions, for example, stress and non-stress, may highlight contrasting attributes and mechanisms. Subsequent to this, comparative analysis of works of art, music and dance may discover common visual, auditory and movement attributes representing the emotions expressed by people, and maybe synesthetic connections between the arts modalities. An additional area where these models could reveal important empirical data is in the implications of image creation by marginal groups in society, trying to understand the ethno-cultural factor in response to art. For example, it would be interesting to explore minority ethnic groups for the mechanisms of image creation of their sense of well-being, social affiliation, coherence and civic engagement.
2. Studies related to the clinical setting for discovering ‘behavioral markers’; i.e., construction/creation and social interaction metrics to predict treatment progress and outcome. These will utilize the clinical setting model. To date, changes in client behavior are recognized intuitively and qualitatively by the therapist as ‘moments-of-change’ or ‘turning-points’ [32, 80]. Using our approach, comparing sessions for an individual patient or between a collective of patients will become possible. We may thus reveal significant markers that are universal to all or most patients, or to some specific groups thereof. Likewise, typical markers per population segment can be discovered, such as per age group, gender, illness.
3. Optimization of arts-based approaches based on the studies in *(i)* and *(ii)*. These may help attune an arts-based intervention; i.e., optimizing the choice of specific arts modality per patient's characteristics, such as, illness, demographic factor. As examples, we wish to be able to provide answers to questions like these: Are the therapeutic effects of art intervention most advantageous for women with Parkinson or with PTSD (posttraumatic stress disorder)? Do autistic children benefit the most from music therapy compared to other forms of arts therapy?

#### Documentation Module

We wish to facilitate and create standardization in the reporting of patient cases and behavioral patterns, in session comparisons and in the documentation, retrieval and sharing of information. An appropriate formal language will be devised to represent the dynamics of the clinical sessions' domain. Examples of preliminary graphical notation for music therapy sessions can be found in [81] and [55]. These and our current textual and visual reports of the dynamics in art sessions (Figs 6, 7, 9, 10 and S5, S6, S7, S8) will be further developed into an automated or semi-automated domain-specific language. Once an agreed-upon language is introduced into a profession it enables ever-growing opportunities of communication and understanding between specialists and communities of the domain’s relevant fields.

The overall developmental approach of our technology is gradual, modular and incremental, and follows the system’s growing complexity, and is thus beneficial for on-going real-world studies throughout. Our Statecharts modeling methodology facilities this by exercising the notion of hierarchy and concurrency not only in the visual formalism itself, but also in the realization of the technology. We plan our empirical infrastructure to allow both intra-/local-/micro- analysis, where the focus is on specific moments within an arts-based session dynamics, and inter-/global-/macro-analysis, with reference to wider perspectives, across sessions, individuals and collectives. This might also lead to an additional research avenue, in which the behavioral results we generate (e.g., gender and age difference in the process of art making) are mapped to bio-neural mechanisms, such as brain activity [82]. We thus believe the approach has the potential of providing a significant boost to arts associated fields.

## Supporting Information

S1 Fig Preliminary models of the therapist and patient. The **Client** (patient) and **ArtTherapist** concurrent (orthogonal) state entities and their auditory and bodily states therein. These are part of the therapy room hierarchy of states originating, for example, in Fig 2 and in the top panel of Fig 3.
(TIF)

S2 Fig A preliminary model of the music session dynamics. (***Top Panel***) The **Music_Work** model is a state in the music room parallel to other states there; i.e., the **Client** (patient) and **Music_Therapist**. (***Bottom Panel***) The music work’s creator can be in one of three states: **Playing**, **Selecting** instruments or **Idle**. These are further decomposed as can be seen in the figure.
(TIF)

S3 Fig Drawing media for the artwork study. (**A**) The tablet’s user interface customized for the participants to draw their art creation. (**B**) Displaying diverse media features of colors, textures, mixing, blending and erasing.
(TIF)

S4 Fig The participants’ instruction protocol for the artwork study. (TIF)

S5 Fig Quantification of an art making process (a positive feeling). The textual report of the creation process dynamics of an artwork imaging positive feeling; the visual report is in Fig 9.
(TIF)

S6 Fig Quantification of an art making process (a negative feeling). The textual report of the creation process dynamics of an artwork imaging negative feeling; the visual report is in Fig 10.
(TIF)

S7 Fig Visualization of an artwork construction dynamics (a positive feeling). The visual report of an artwork imaging a positive feeling, exemplifying notable circular motions of blending in of white.
(TIF)

S8 Fig Visualization of an artwork construction dynamics (a house-tree-person). The visual report of an artwork imaging the narrative of house-tree-person, exemplifying meaningful erasures and image changes.
(TIF)

S9 Fig Gender colors and tools use. (**A**) (*Upper left panel*) Use of colors depicted as percentage of drawing time in all three major drawing tasks for females, males, and all participants. (*Bottom left panel*) Use of colors in the house-tree-person task. (*Upper right panel*) Use of colors in the negative feeling task. (*Bottom right panel*) Use of colors in the positive feeling task. (**B**) Use of tools as percentage of drawing time. Panels as in (A). (**C**) (*Left panel*) Use of color palette displayed as percentage of total color choices for females, males and all participants, per drawing tasks and them all. (*Right panel*) Use of tools displayed as percentage of total tool choices for collectives, as in the left panel. *p < 0.05, **p < 0.01.
(TIF)

S10 Fig Collective colors use. Collective use of colors for all participants as percentage of drawing time. (***Upper left panel***) Color use in all three major drawing tasks. (***Bottom left panel***) Color use in the house-tree-person task. (***Upper right panel***) Color use in the negative feeling task. (***Bottom right panel***) Color use in the positive feeling task.
(TIF)

S11 Fig Collective tools use. Collective use of tools for all participants as percentage of drawing time. (***Upper left panel***) Tool use in all three major drawing tasks. (***Bottom left panel***) Tool use in the house-tree-person task. (***Upper right panel***) Tool use in the negative feeling task. (***Bottom right panel***) Tool use in the positive feeling task.
(TIF)

S12 Fig Calibration drawing task artworks. (***Left column***) Females’ drawings. (***Right column***) Males’ drawings.
(TIF)

S13 Fig Positive feeling drawing task artworks. (***Left column***) Females’ drawings. (***Right column***) Males’ drawings.
(TIF)

S14 Fig Negative feeling drawing task artworks. (***Left column***) Females’ drawings. (***Right column***) Males’ drawings.
(TIF)

S15 Fig House-Tree-Person drawing task artworks. (***Left column***) Females’ drawings. (***Right column***) Males’ drawings.
(TIF)

## References

[1] Nainis N, Paice J, Ratner J, Wirth J, Lai J, Shott S (2006) Relieving symptoms in cancer: innovative use of art therapy. J Pain Symptom Manage 31: 162–169. 16488349 10.1016/j.jpainsymman.2005.07.006

[2] Bar-Sela G, Atid LDS, Gabay N, Epelbaum R (2007) Art therapy improved depression and influenced fatigue level in cancer patients on chemotherapy. Psycho-Oncol 16: 980–984. 10.1002/pon.1175 17351987

[3] Thyme K, Sundin E, Wiberg B, Oster I, Astrom S, Lindh J (2009) Individual brief art therapy can be helpful for women with breast cancer: a radomized controlled clinical study. Palliat Support Care 7: 87–95. 10.1017/S147895150900011X 19619378

[4] Tusek DL, Cwynar R, Cosgrove DM (1999) Effect of guided imagery on length of stay, pain and anxiety in cardiac surgery patients. J Cardiovasc Manage 10: 22–28. 10557909

[5] Czamanski-Cohen J, Sarid O, Huss E, Ifergane A, Niego L, Cwikel J (2014) CB-ART—The use of a hybrid cognitive behavioral and art based protocol for treating pain and symptoms accompanying coping with chronic illness. Arts Psychother 41: 320–328.

[6] McNiff S (1992) Art as medicine, Boston: Shambhala Press. 235 p.

[7] Richardson P, Jones K, Evans C, Stevens P, Rowe A (2007) Exploratory RCT of art therapy as an adjunctive treatment in schizophrenia. J Ment Health 16: 483–491.

[8] Dileo C (2006) Effects of music and music therapy on medical patients: a meta-analysis of the research and implications for the future. J Soc Integr Oncol 4: 67–70. 19442338 10.2310/7200.2006.002

[9] Burns S, Harbuz M, Hucklebridge F, Bunt A (2001) A pilot study into the therapeutic effects of music therapy at a cancer help center. Altern Ther Health Med 7:48–57. 11191042

[10] Guzzetta C (1989) Effects of relaxation and music therapy in a coronary care unit with presumptive acute myocardial infaction. Heart Lung 18: 609–616. 2684920

[11] Dassa A, Amir D (2014) The role of singing familiar songs in encouraging conversation among people with middle to late stage Alzheimer's disease. J Music Ther 51: 131–153. 10.1093/jmt/thu007 25013944

[12] Pacchetti C, Mancini F, Aglieri R, Fundaro C, Martignoni E, Nappi G (2000) Active music therapy in Parkinson's disease: an integrative method for motor and emotional rehabilitation. Psychosom Med 62: 386–39. 10845352 10.1097/00006842-200005000-00012

[13] Hilliard R (2003) The effects of music therapy on the quality and length of life of people diagnosed with terminal cancer. J Music Ther 40:113–137. 14505443 10.1093/jmt/40.2.113

[14] Gold C, Solli H, Kruger V, Lie S (2009) Dose-response relationship in music therapy for people with serious mental disorders: systematic review and meta-analysis. Clin Psychol Rev 29: 193–207. 10.1016/j.cpr.2009.01.001 19269725

[15] Sandel S, Judge J, Landry N, Farina L, Ouellette R, Majczak M (2005) Dance and movement program improve quality-of-life measures in breat cancer survivors. Cancer Nurs 28: 301–309. 16046894 10.1097/00002820-200507000-00011

[16] Koch S, Kunz T, Lykou S, Cruz R (2014) Effects of dance movement therapy and dance on health-related psychological outcomes: a meta-analysis. Arts Psychother 41: 46–64. 10.3389/fpsyg.2019.01806 PMC6710484 31481910

[17] Kiepe MS, Stockigt B, Keil T (2012) Effects of dance therapy and ballroom damces on physical and mental illnesses: A systematic review. Arts Psychother 39: 404–411.

[18] Graham J, Lobel M, Glass P, Lokshina I (2008) Effects of written anger expression in chronic pain patients: making meanng from pain. J Behav Med 31: 201–212. 10.1007/s10865-008-9149-4 18320302

[19] Malchiodi C (2012) Art therapy and healthcare, New York: Guilford Publications. 388p.

[20] Italia S, Favara‐Scacco C, Di Cataldo A, Russo G (2008) Evaluation and art therapy treatment of the burnout syndrome in oncology units. Psycho‐Oncol 17:676–680. 10.1002/pon.1293 17992704

[21] Huss E, Sarid O (2014) Visually transforming artwork and guided imagery as a way to reduce work related stress: A quantitative pilot study. Arts Psychother 41: 409–412.

[22] Belkofer CM, Van Hecke AV, Konopka LM (2014) Effects of drawing on alpha activity: A Quantitative EEG study with implications for art therapy. Art Ther 31: 61–68.

[23] Chanda ML, Levitin DJ (2013) The neurochemistry of music. Trends Cogn Sci 17: 179–193. 10.1016/j.tics.2013.02.007 23541122

[24] Lindblad F, Hogmark Å, Theorell T (2007) Music intervention for 5th and 6th graders—effects on development and cortisol secretion. Stress Health 23: 9–14.

[25] Smolen D, Topp R, Singer L (2002) The effect of self-selected music during colonoscopy on anxiety, heart rate, and blood pressure. App Nurs Res 15: 126–136. 10.1053/apnr.2002.34140 12173164

[26] Kumar AM, Tims F, Cruess DG, Mintzer MJ, Ironson G, Loewenstein D, et al (1999) Music therapy increases serum melatonin levels in patients with Alzheimer's disease. Alter Ther Health Med 5: 49–57. 10550905

[27] Batson G, Migliarese SJ, Soriano C, Burdette JH, Laurienti PJ (2014) Effects of Improvisational Dance on Balance in Parkinson's Disease: A Two-Phase fMRI Case Study. Phys Occup Ther Geriatr 32: 188–197.

[28] Junge MB, Assawa PP (1994) A history of art therapy in the United States Mundelein, IL: American Art Therapy Association.

[29] Devlin B (2006) The art of healing and knowing in cancer and palliative care. Int J Palliative Med 12: 16–19. 10.12968/ijpn.2006.12.1.20391 16493300

[30] Stuckey HL, Nobel J (2010) The connection between art, healing, and public health: A review of current literature. American Journal of Public Health 100: 254–263. 10.2105/AJPH.2008.156497 20019311 PMC2804629

[31] Perruza N, Kinsella EA (2010) Creative arts occupations in therapeutic practice: a review of the literature. Br J Occup Ther 73: 261–268.

[32] Bell B (2002) Moments of change in art therapy process. Arts Psychother 29: 79–92.

[33] McLean CL (2014) Creative arts in humane medicine Brush Education (University of Toronto Press). 240 p.

[34] Jones P (2005) The arts therapies: A revolution in healthcare New York: Brunner- Routledge. 312 p.

[35] Cruz RF, Feder B (2013) The art and science of evaluation in art therapies, 2nd ed., Charles C Thomas Pub Ltd. 396 p.

[36] Cohen BM, Hammer JS, Singer S (1988) The Diagnostic Drawing Series: a systematic approach to art therapy evaluation and research. Arts Psychother 15:11–21.

[37] Gantt LM (2001) The Formak Elements Art Therapy Scale: a measurement system for global variables in art. Art Ther 18: 51–55.

[38] Monti DA, Peterson C, Kunkel EJS, Hauck WW, Pequignot E, Rhodes L, et al (2006) A randomized, controlled trial of mindfulness‐based art therapy (MBAT) for women with cancer. Psycho-Oncol 15:363–373. 10.1002/pon.988 16288447

[39] Chapman L, Morabito D, Ladakakos C, Schreier H, Knudson MM (2001) The effectiveness of art therapy interventions in reducing post traumatic stress disorder (PTSD) symptoms in pediatric trauma patients. Art Ther 18: 100–104.

[40] Kim S (2010) A computer system for the analysis of color-related elements in art therapy assessment: Computer_Color-Related Elements Art Therapy evaluation System (C_CREATS). Arts Psychother 37: 378–386.

[41] Kruk KA, Aravich PF, Deaver SP, deBeus R (2014) Comparison of brain activity during drawing and clay sculpting: A preliminary qEEG sudy. Art Ther 31: 52–60.

[42] Huss E (2009) 'A coat of many colors'—Towards an integrative multilayered model of art therapy. Arts Psychother 36: 154–160.

[43] Allen P (1995) Art is a way of knowing: A guide to self-knowledge and spiritual fulfillment through creativity Boston: Shambhala Press. 224 p.

[44] Dalley T (2009) Art as therapy: an introduction to the use of art as a therapeutic technique New-York: Tavistock/Routledge. 240 p.

[45] Sarid O, Huss E (2011) Image formation and image transformation. Arts Psychother 38: 252–255.

[46] Harel D (1987) Statecharts: A visual formalism for complex systems. Sci Comp Program 8: 231–274.

[47] Bittman BB, Berk LS, Felten DL, Westengard J, Simonton OC, Pappas J, et al (2001) Composite effects of group drumming music therapy on modulation of neuroendocrine-immnue parameters in normal subjects. Alt Ther Health Med 7: 38–47. 11191041

[48] Harel D, Pnueli A (1985) On the development of reactive systems. In: Apt K, editor Logics and models of concurrent systems. New York: Springer-Verlag, pp. 477–498.

[49] Harel D (1988) On visual Formalisms. Comm Assoc Comput Mach 31: 514–530.

[50] Drusinsky D, Harel D (1987) Using Statecharts for hardware description. In: Proc. IEEE CAD Conf., Santa Clara, CA, November.

[51] Douglass BP, Harel D, Trakhtenbrot M (1998) Statecharts in use: Structured analysis and object-orientation (Lectures on Embedded Systems). In: Vaandrager F, Rozenberg G, editors. Lect Notes Comp Sci, Springer-Verlag 1494: 368–394.

[52] Kam N, Cohen IR, Harel D (2001) The immune system as a reactive system: modeling T cell activation with Statecharts. In: Proc. Visual Languages and Formal Methods (VLFM'01), part of IEEE Symp. on Human-Centric Computing (HCC'01).

[53] Sobolev B, Harel D, Vasilakis C, Levy A (2008) Using the Statecharts paradigm for simulation of patient flow in surgical care. Health Care Manag Sci 11: 79–86. 18390170 10.1007/s10729-007-9026-7

[54] Swerdlin N, Cohen IR, Harel D (2008) The lymph node B cell immune response: dynamic analysis in-silico. In: Proceedings of the IEEE (special issue on Computational System Biology).

[55] Gilboa A, Bensimon M (2007) Putting clinical process into image:A method for visual representation of music therapy sessions. Music Ther Perspect 29: 32–42.

[56] Mathworks—Simulink- simulation and model-based design. Available: http://www.mathworks.com/products/simulink. Accessed 1 January 2015.

[57] Mathworks—MATLAB—the language of technical computing. Available: http://www.mathworks.com/products/matlab. Accessed 1 January 2015.

[58] Mathworks—Stateflow—model and simulate decision logic using state machine and flow charts. Available: http://www.mathworks.com/products/stateflow. Accessed 1 January 2015.

[59] Harel D, Kugler H (2004) The Rhapsody semantics of Statecharts (or, On the executable core of the UML). In: Ehrig H, et al editors. Integration of Software Specification techniques for Applications in Engineering. Lect Notes Comp Sci, Springer-Verlag 3147: 325–354.

[60] Orr P (2012) Technology use in art therapy practice: 2004 and 2011 comparison. Arts Psychother 39:234–238.

[61] Ambient Design—ArtRage4. Available: http://www.artrage.com/artrage-4/. Accessed 1 January 2015.

[62] Mathworks—Statistics Toolbox—analyse and model data using statistics and machine learning. Available: http://www.mathworks.com/products/statistics/. Accessed 1 January 2015.

[63] Silver R (2005) Aggression and depression assessed through art New York: Brunner and Routledge. 232 p.

[64] Mason J (2002) Qualitative researching. London: Sage Publications. 224 p.

[65] Ullman S (1996) High-level Vision: object recognition and visual cognition, Cambridge: The MIT Press. 432 p.

[66] Adams RBJ (2011) The science of social vision Oxford University Press. 504 p.

[67] Fridin M, Barilya A, Schechtman E, de Gelder B, Tamar F (2009) Computational model and human perception of emotional body language (EBL). In: Proceeding of the Symposium on Mental States, Emotions and their Embodiment. Edinburgh, Scotland.

[68] Everingham M, Eslami SMA, Van Gool L, Williams CKI, Winn J, et al. (2014) The PASCAL Visual Object Classes Challenge—a Retrospective. Int J Comp Vision: 1–39.

[69] OpenCV—Open Source Computer Vision Library. Available: http://opencv.org/. Accessed 1 January 2015.

[70] Ekman P (1999) Basic Emotions In: Dagliesh T, Power T, editors. Handbook of Cognition and Emotion. Sussex, UK:John Wiley & Sons pp. 301–320.

[71] Mathworks—Computer Vision System Toolbox—design and simulate computer vision and video processing systems. Available: http://www.mathworks.com/products/computer-vision/. Accessed 1 January 2015.

[72] Mathworks—Image Processing Toolbox—perform image processing, analysis, and algorithm development. Available: http://www.mathworks.com/products/image/. Accessed 1 January 2015.

[73] Marsella S, Gratch J (2014) Computationally modeling human emotion. Commun. ACM 57: 56–67.

[74] Reuderink B, Poel M, Truong K, Poppe R, Pantic M (2008) Decision-Level Fusion for Audio-Visual Laughter Detection. In: MLMI 2008, Lect Notes Comp Sci 5237:137–148.

[75] Eshkol N, Wachmann A (1958) Movement Notation. London: Weidenfeld and Nicolson. 203 p.

[76] Hutchinson A (1987) Labanotation: The system of analyzing and recording movement 3rd Ed. New York: Routledge/Theatre Arts Books. 528 p.

[77] Benesh R, Benesh J (1983) Reading dance: The birth of choreology Souvenir Press. 139 p.

[78] Sandak B, Nussinov R, Wolfson HJ (1998) A method for biomolecular structural recognition and docking allowing conformational flexibility. J Comp Biol 5:631–654. 10.1089/cmb.1998.5.631 10072081

[79] Sandak B, Wolfson HJ, Nussinov R (1998) Flexible docking allowing induced fit in proteins: Insights from an open to closed conformational isomers. Proteins: Struc, Func & Genet 32: 159–174. 9714156

[80] Greenberg LS (1994) The investigations of change: Its measurements and explanations In: Reassessing psychotherapy research. New York: The Guilford Press, pp. 114–143.

[81] Bergstrom-Nielsen (2009) Graphic notation in music therapy: A discussion of what to notate in graphic notation, and how, In: Approaches: Music Therapy & Special Music Education 1: 72–92. 10.3389/fpsyg.2014.00706 25071671 PMC4086404

[82] Bolwerk A, Mack-Andrick J, Lang FR, Dörfler A, Maihöfner C (2014) How art changes your brain: differential effects of visual art production and cognitive art evaluation on functional brain connectivity. PLOS ONE, 9(7): p. e101035 10.1371/journal.pone.0101035 24983951 PMC4077746

