# Supplementary figures and images for: Computational Paradigm to Elucidate the Effects of Arts-Based Approaches and Interventions: Individual and Collective Emerging Behaviors in Artwork Construction
Source: PLoS One. 2015 Jun 10;10(6):e0126467. doi: 10.1371/journal.pone.0126467 (PMC4489499; doi:10.1371/journal.pone.0126467)

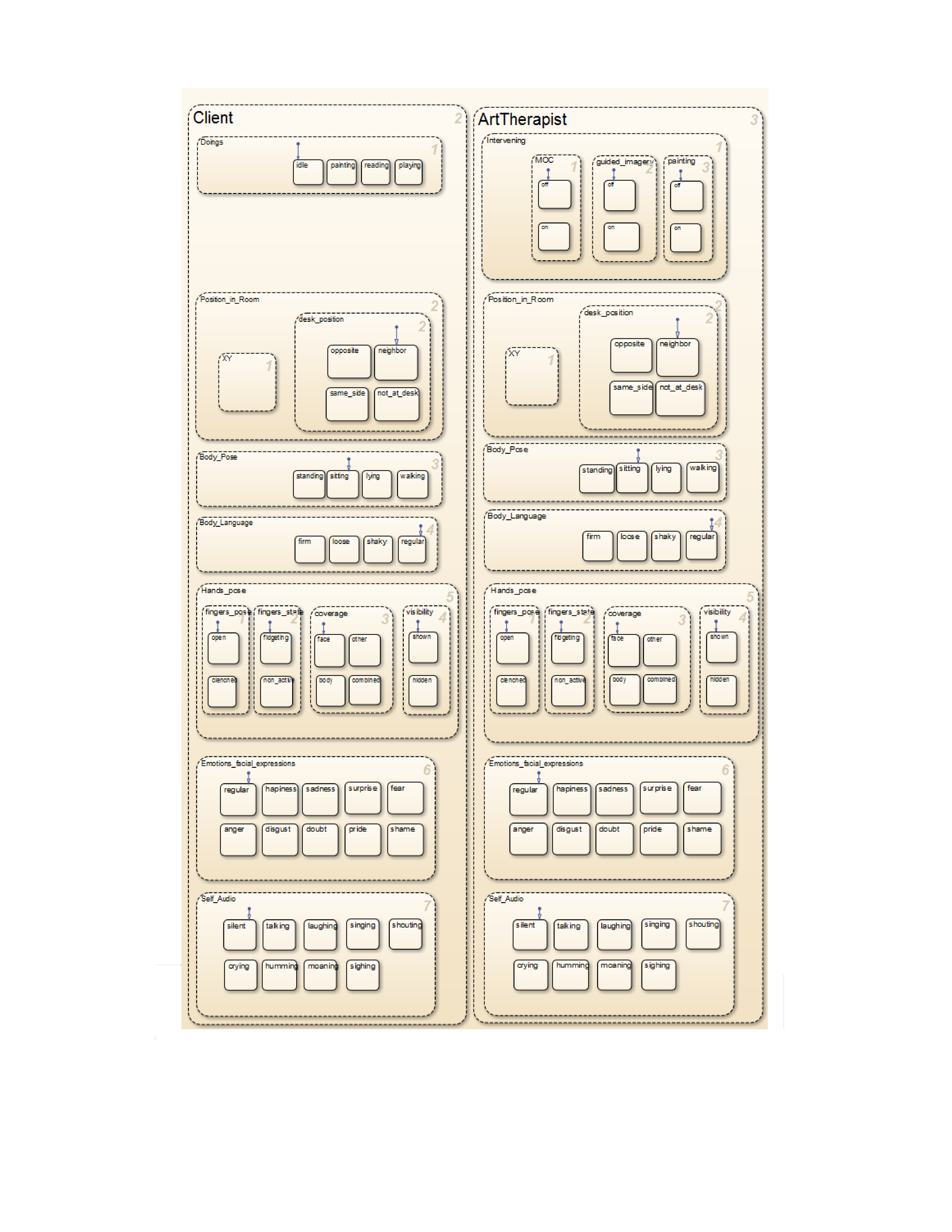

Supplement: S1 Fig — The Client (patient) and ArtTherapist concurrent (orthogonal) state entities and their auditory and bodily states therein. These are part of the therapy room hierarchy of states originating, for example, in Fig 2 and in the top panel of Fig 3. (TIF) [file pone.0126467.s001.tif]

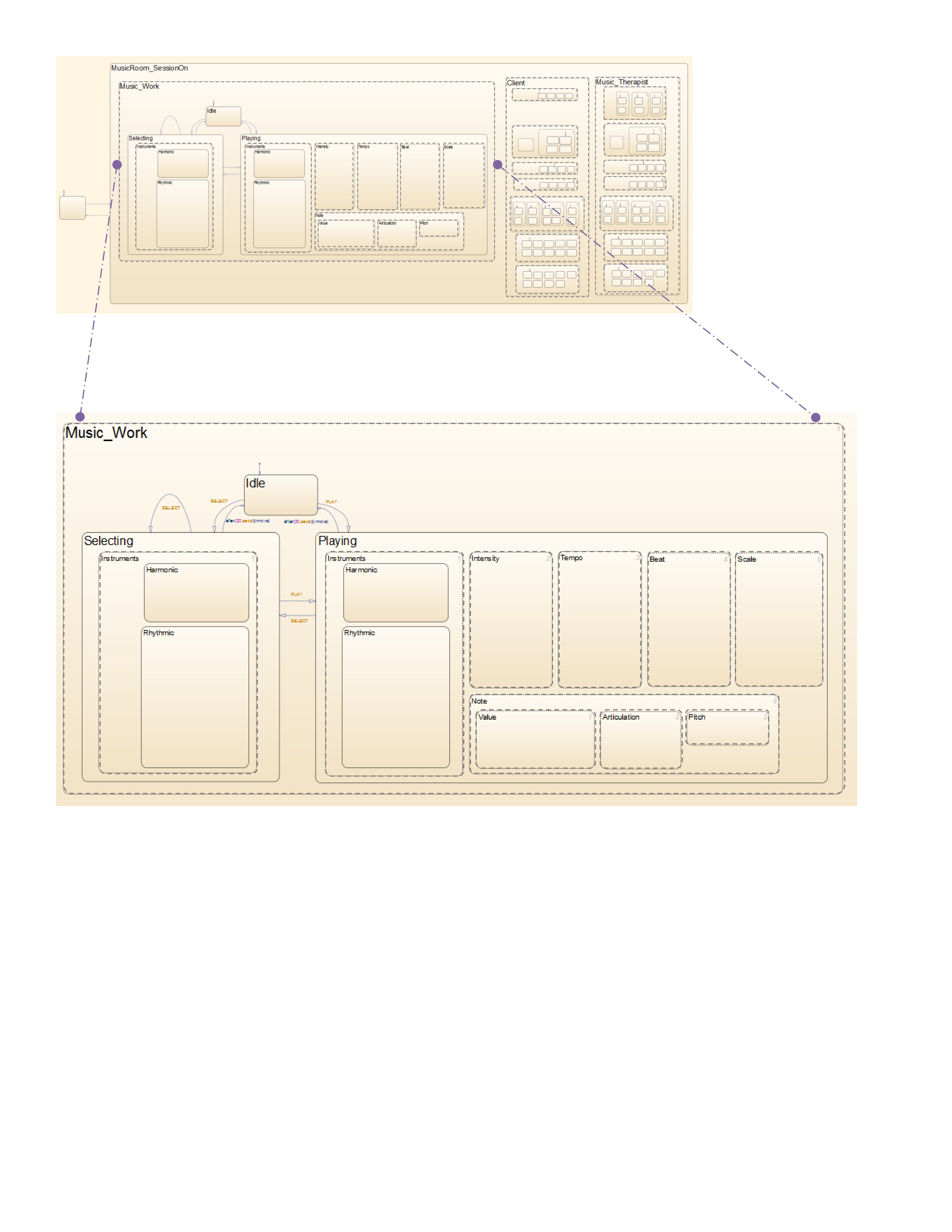

Supplement: S2 Fig — (Top Panel) The Music_Work model is a state in the music room parallel to other states there; i.e., the Client (patient) and Music_Therapist. (Bottom Panel) The music work’s creator can be in one of three states: Playing, Selecting instruments or Idle. These are further decomposed as can be seen in the figure. (TIF) [file pone.0126467.s002.tif]

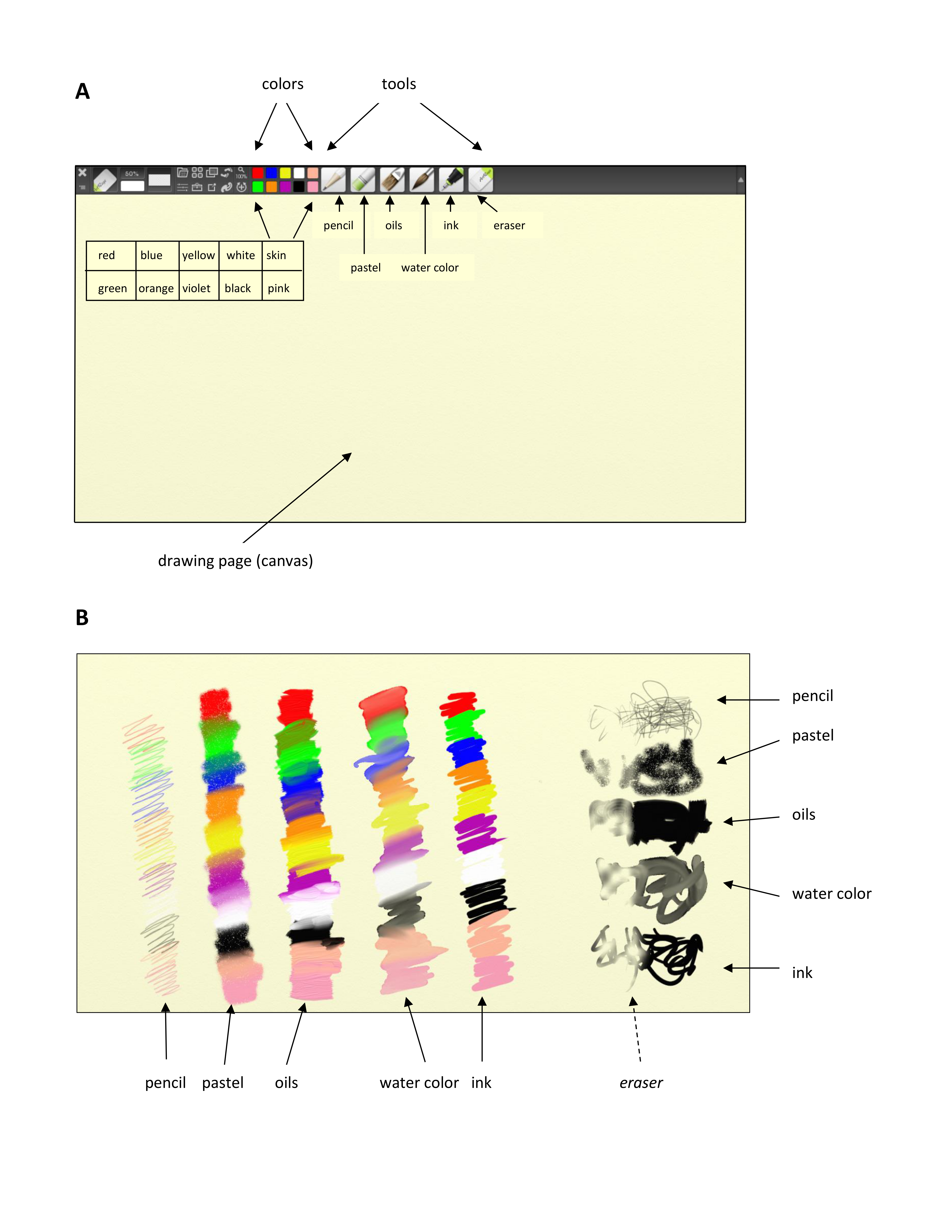

Supplement: S3 Fig — (A) The tablet’s user interface customized for the participants to draw their art creation. (B) Displaying diverse media features of colors, textures, mixing, blending and erasing. (TIF) [file pone.0126467.s003.tif]

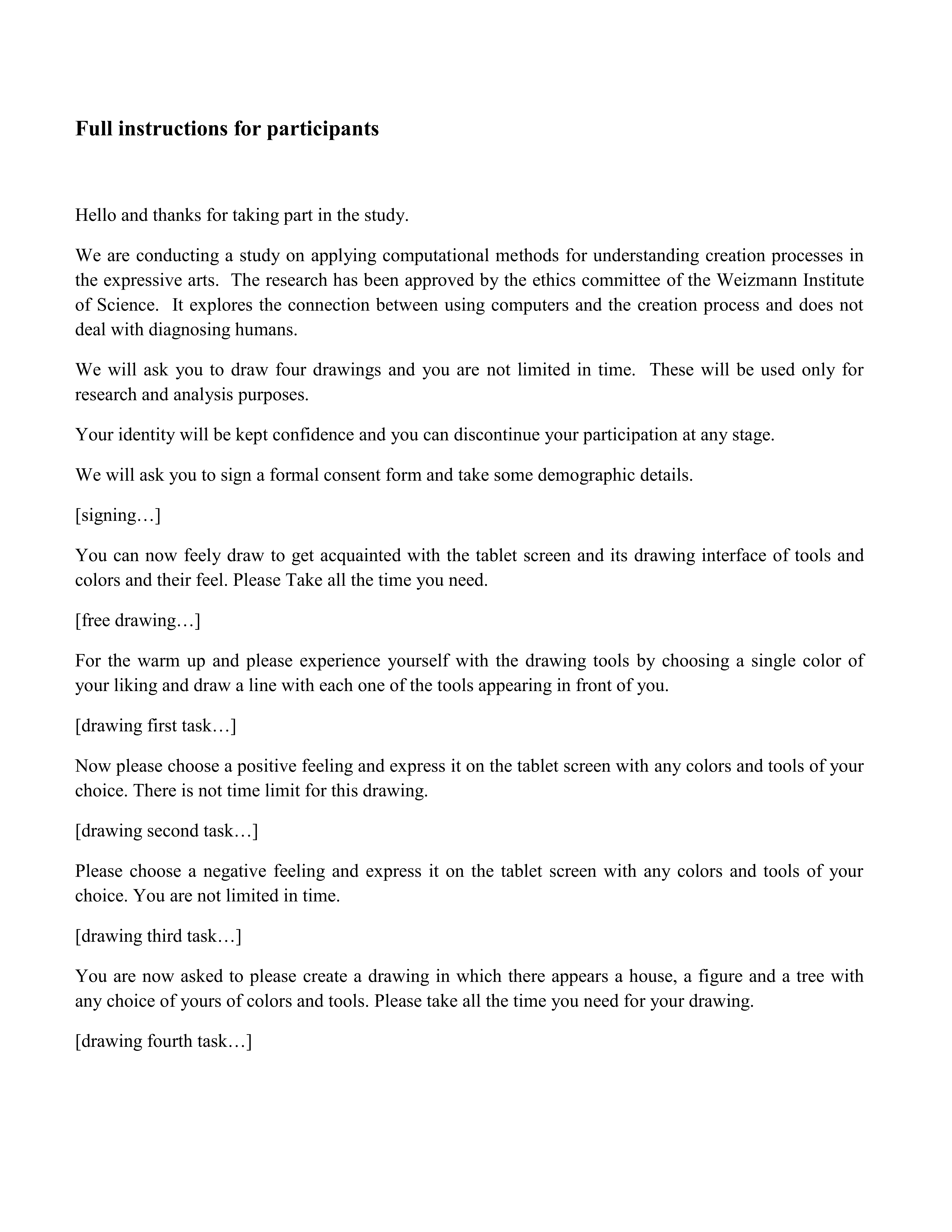

Supplement: S4 Fig — (TIF) [file pone.0126467.s004.tif]

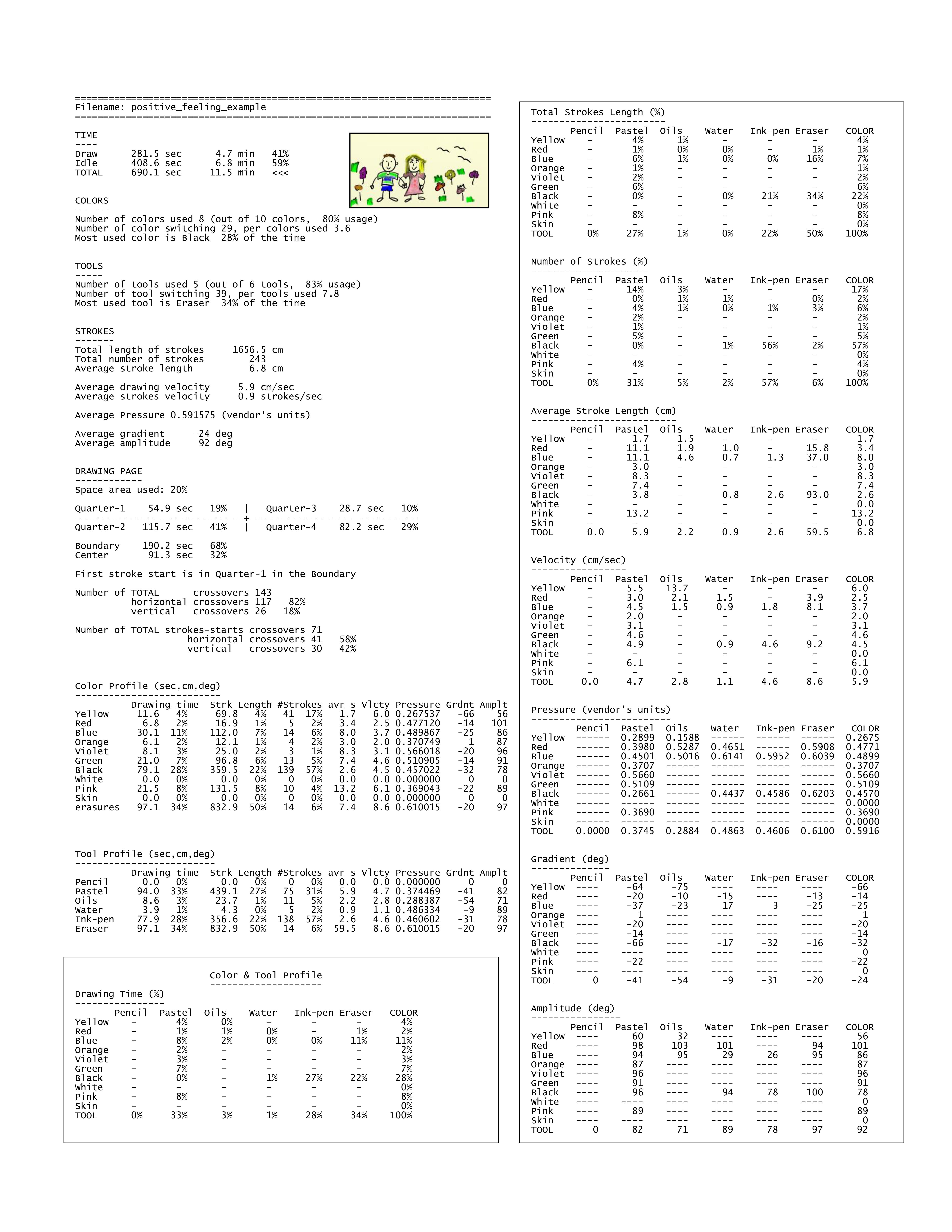

Supplement: S5 Fig — The textual report of the creation process dynamics of an artwork imaging positive feeling; the visual report is in Fig 9. (TIF) [file pone.0126467.s005.tif]

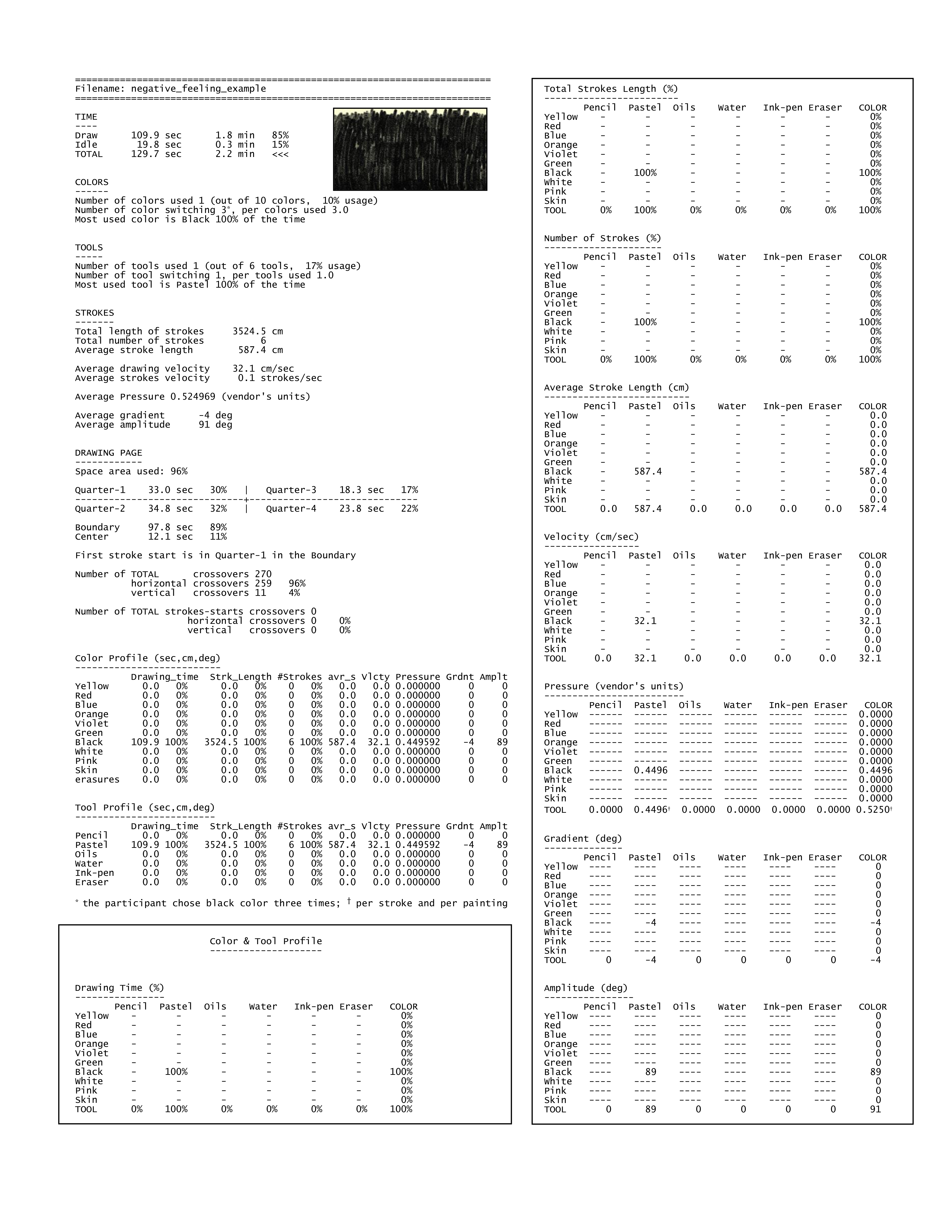

Supplement: S6 Fig — The textual report of the creation process dynamics of an artwork imaging negative feeling; the visual report is in Fig 10. (TIF) [file pone.0126467.s006.tif]

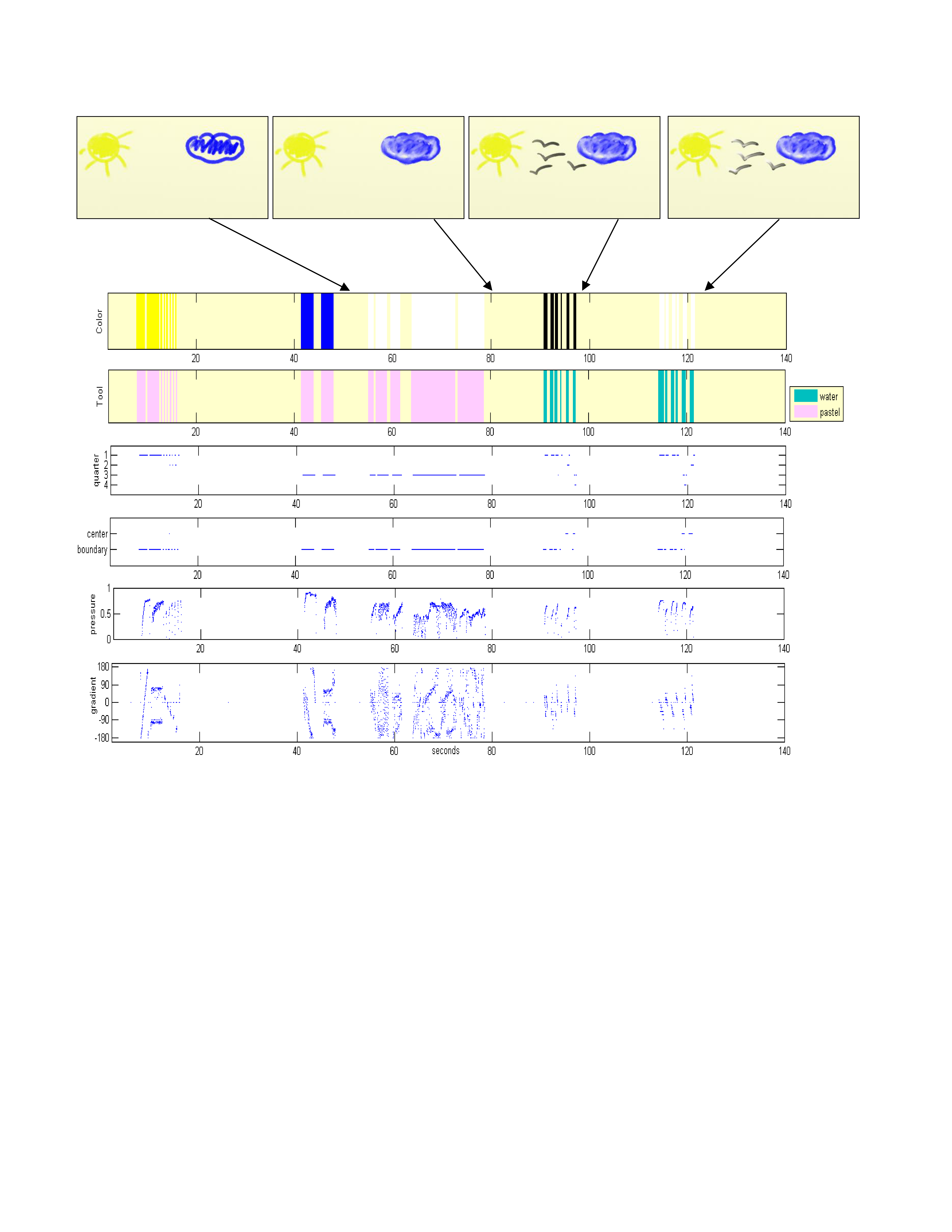

Supplement: S7 Fig — The visual report of an artwork imaging a positive feeling, exemplifying notable circular motions of blending in of white. (TIF) [file pone.0126467.s007.tif]

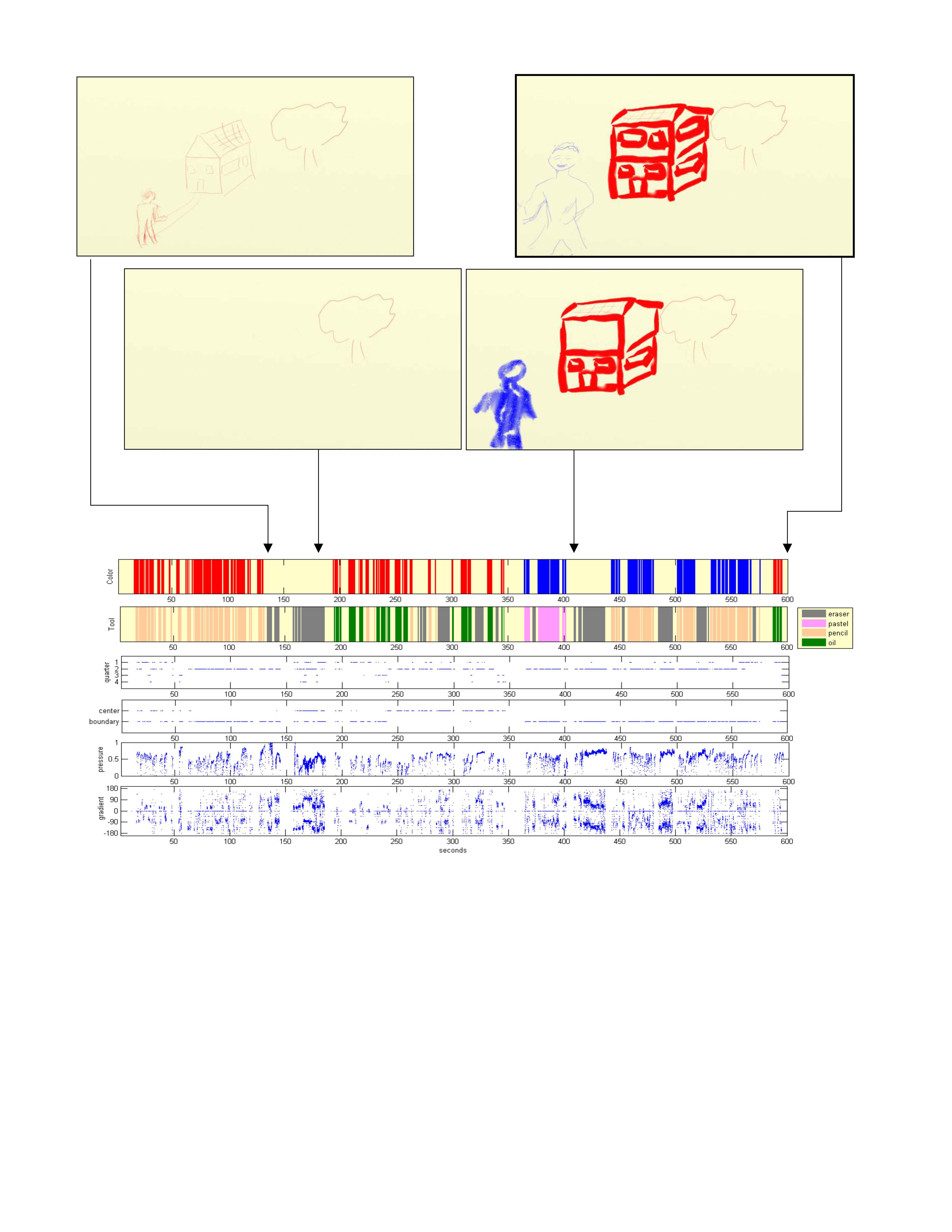

Supplement: S8 Fig — The visual report of an artwork imaging the narrative of house-tree-person, exemplifying meaningful erasures and image changes. (TIF) [file pone.0126467.s008.tif]

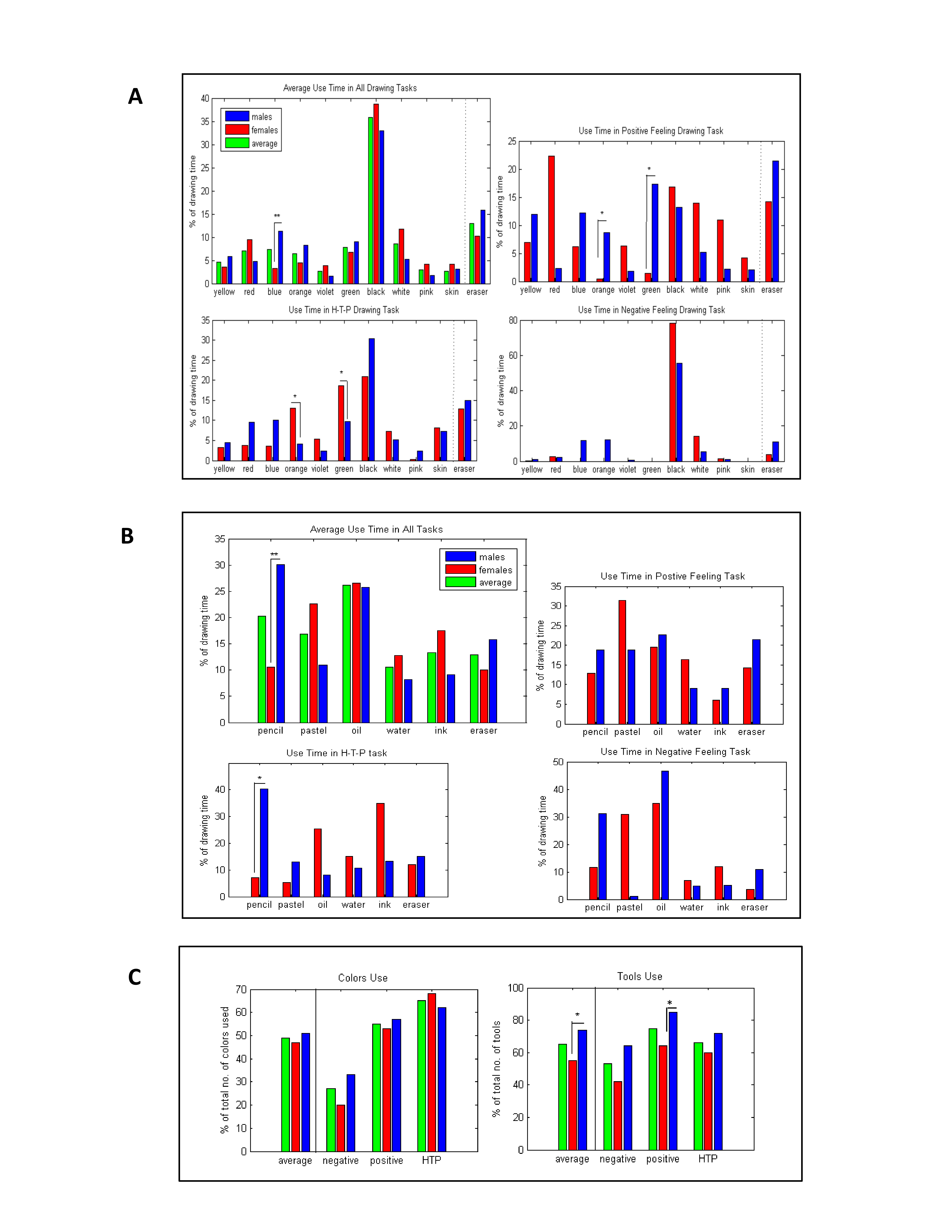

Supplement: S9 Fig — (A) (Upper left panel) Use of colors depicted as percentage of drawing time in all three major drawing tasks for females, males, and all participants. (Bottom left panel) Use of colors in the house-tree-person task. (Upper right panel) Use of colors in the negative feeling task. (Bottom right panel) Use of colors in the positive feeling task. (B) Use of tools as percentage of drawing time. Panels as in (A). (C) (Left panel) Use of color palette displayed as percentage of total color choices for females, males and all participants, per drawing tasks and them all. (Right panel) Use of tools displayed as percentage of total tool choices for collectives, as in the left panel. *p < 0.05, **p < 0.01. (TIF) [file pone.0126467.s009.tif]

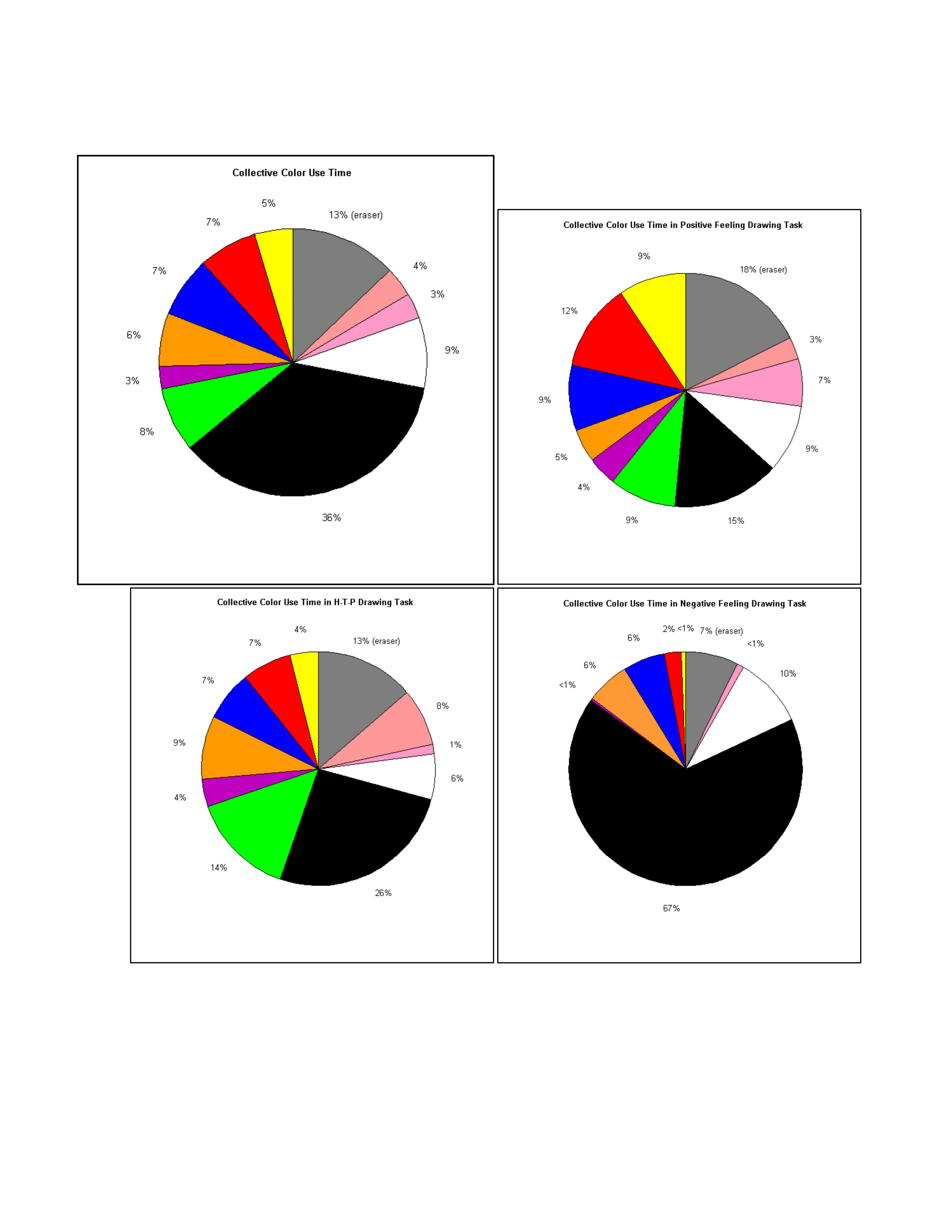

Supplement: S10 Fig — Collective use of colors for all participants as percentage of drawing time. (Upper left panel) Color use in all three major drawing tasks. (Bottom left panel) Color use in the house-tree-person task. (Upper right panel) Color use in the negative feeling task. (Bottom right panel) Color use in the positive feeling task. (TIF) [file pone.0126467.s010.tif]

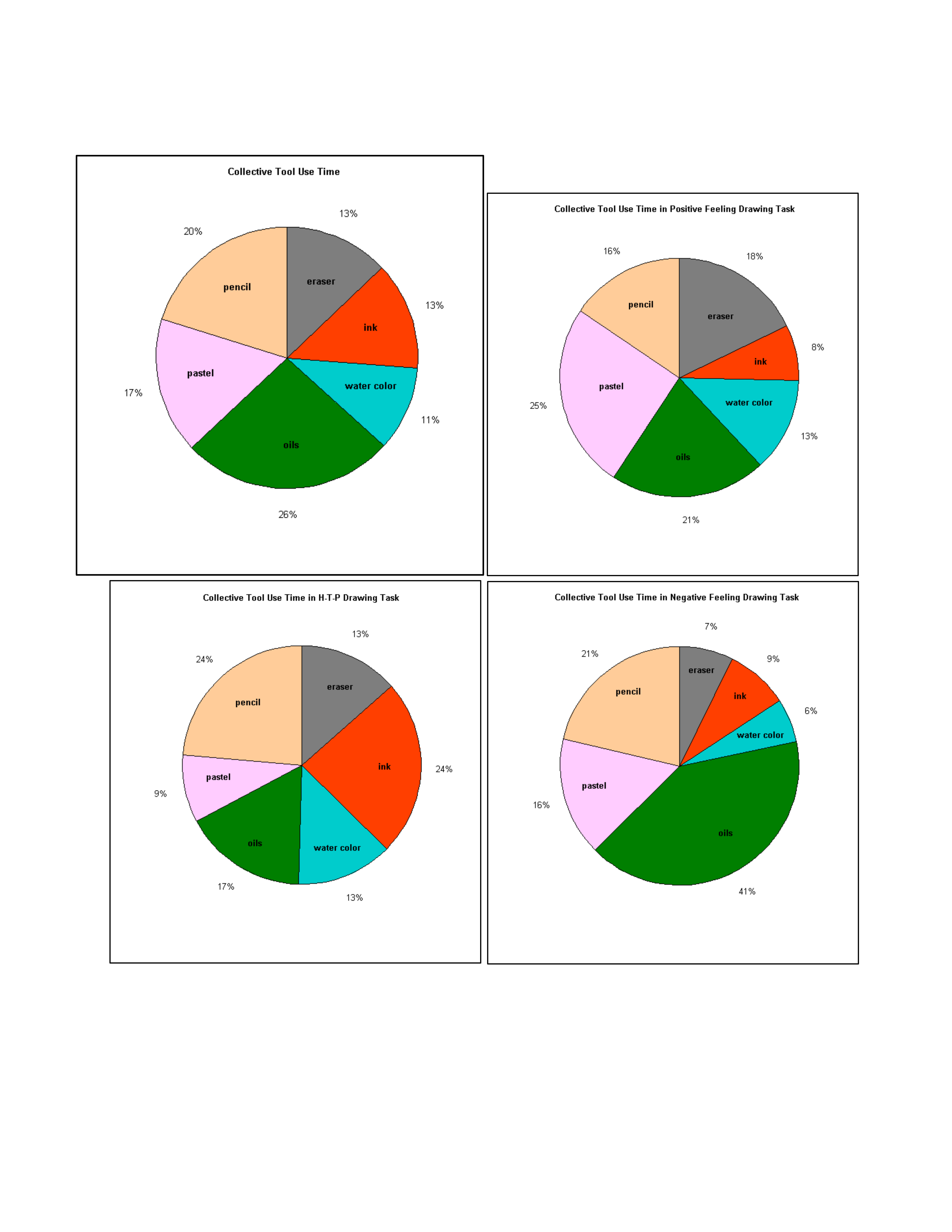

Supplement: S11 Fig — Collective use of tools for all participants as percentage of drawing time. (Upper left panel) Tool use in all three major drawing tasks. (Bottom left panel) Tool use in the house-tree-person task. (Upper right panel) Tool use in the negative feeling task. (Bottom right panel) Tool use in the positive feeling task. (TIF) [file pone.0126467.s011.tif]

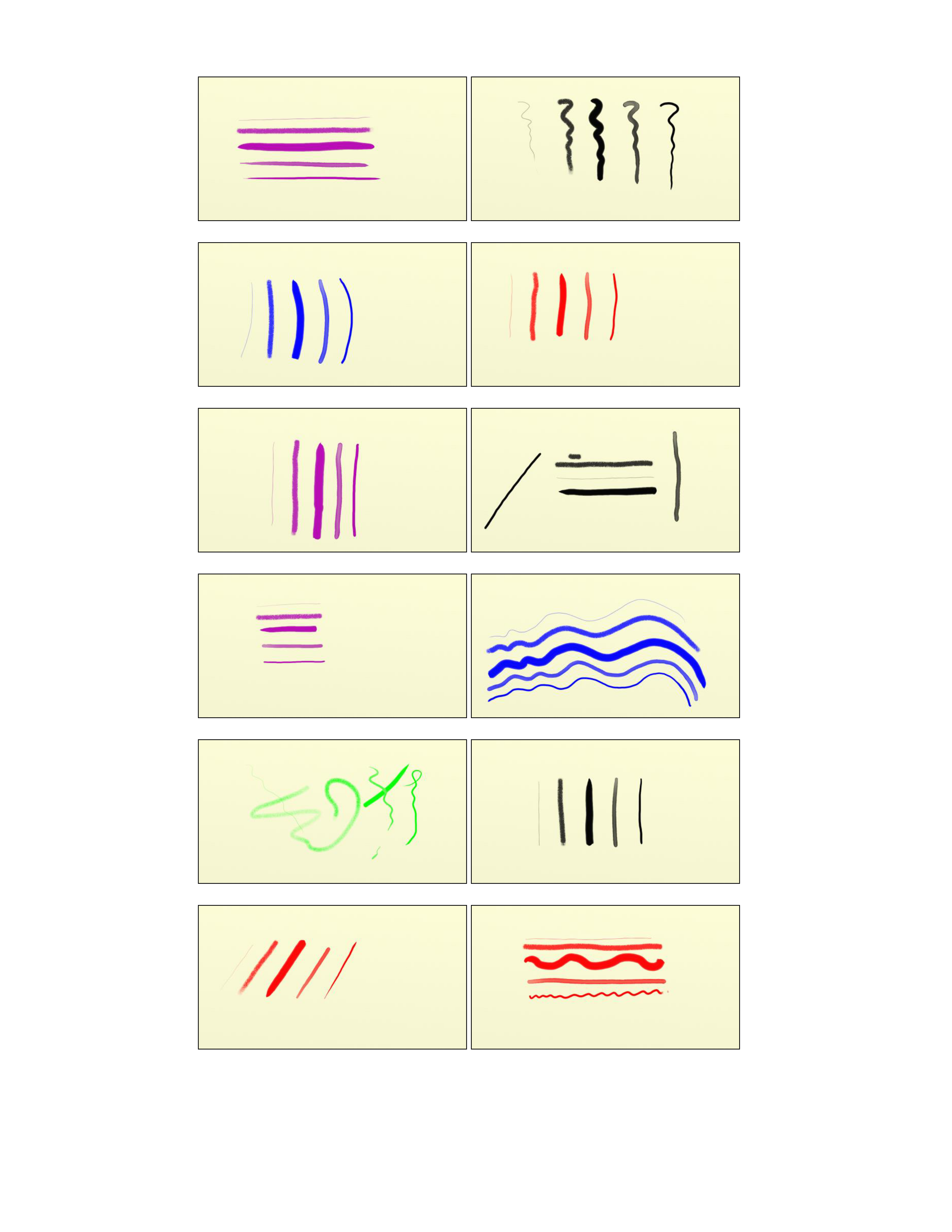

Supplement: S12 Fig — (Left column) Females’ drawings. (Right column) Males’ drawings. (TIF) [file pone.0126467.s012.tif]

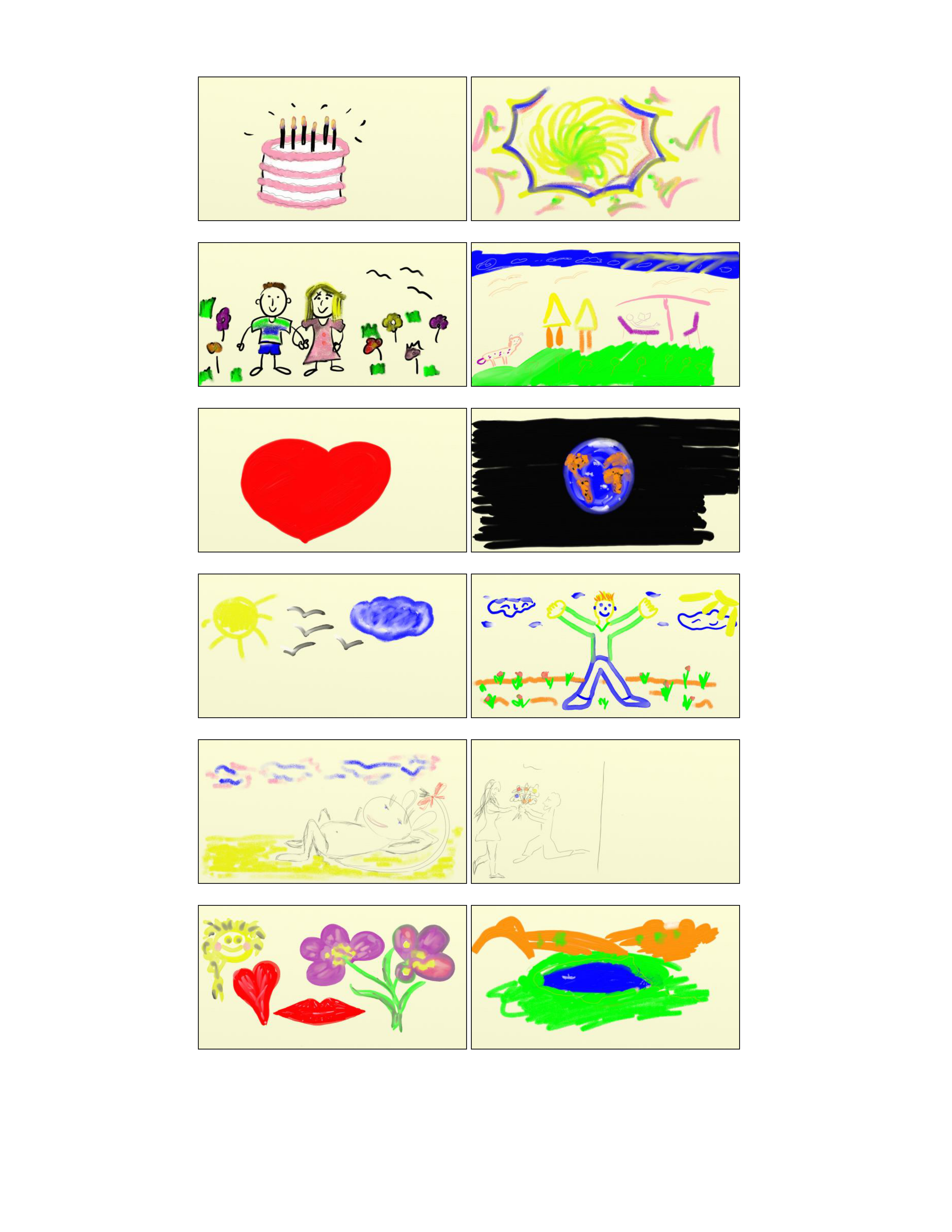

Supplement: S13 Fig — (Left column) Females’ drawings. (Right column) Males’ drawings. (TIF) [file pone.0126467.s013.tif]

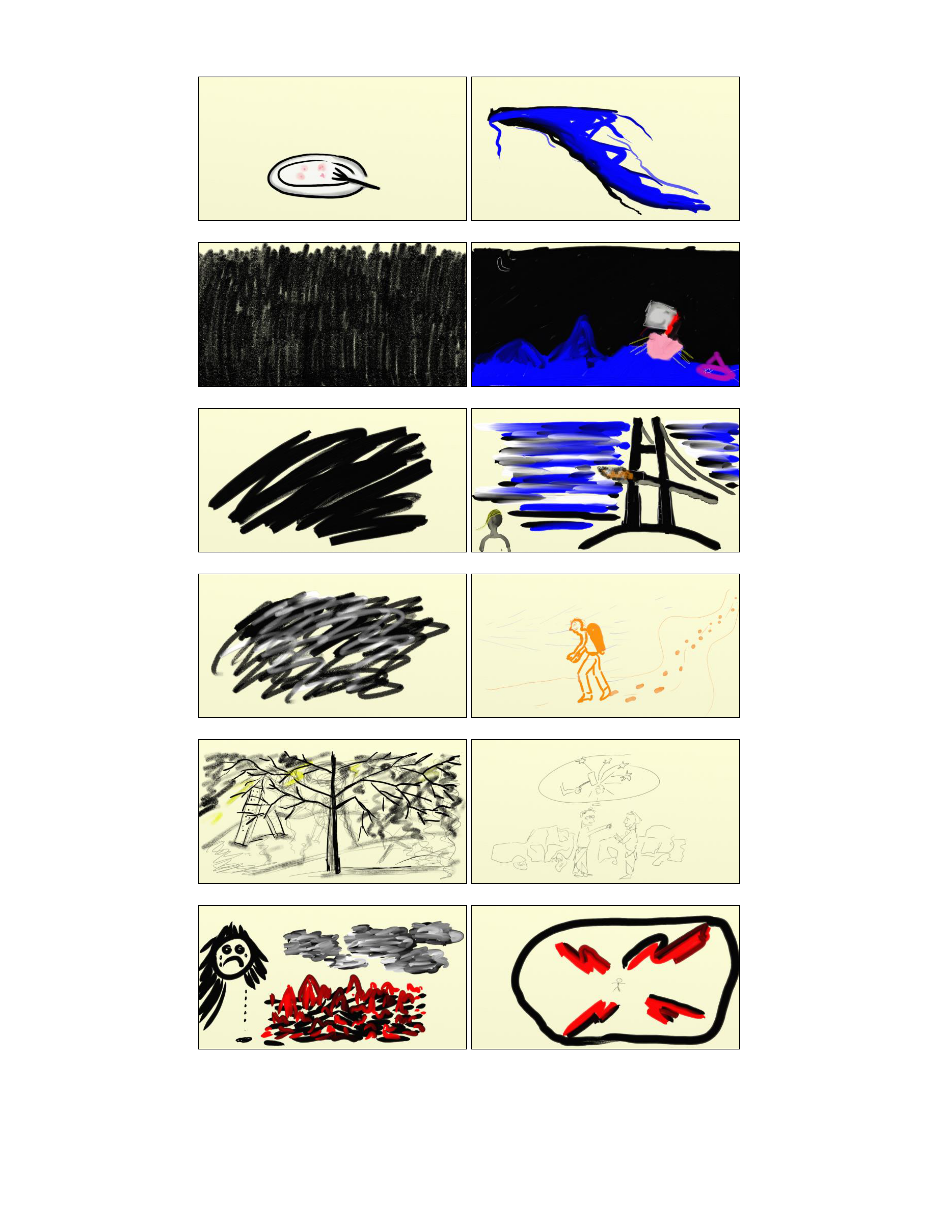

Supplement: S14 Fig — (Left column) Females’ drawings. (Right column) Males’ drawings. (TIF) [file pone.0126467.s014.tif]

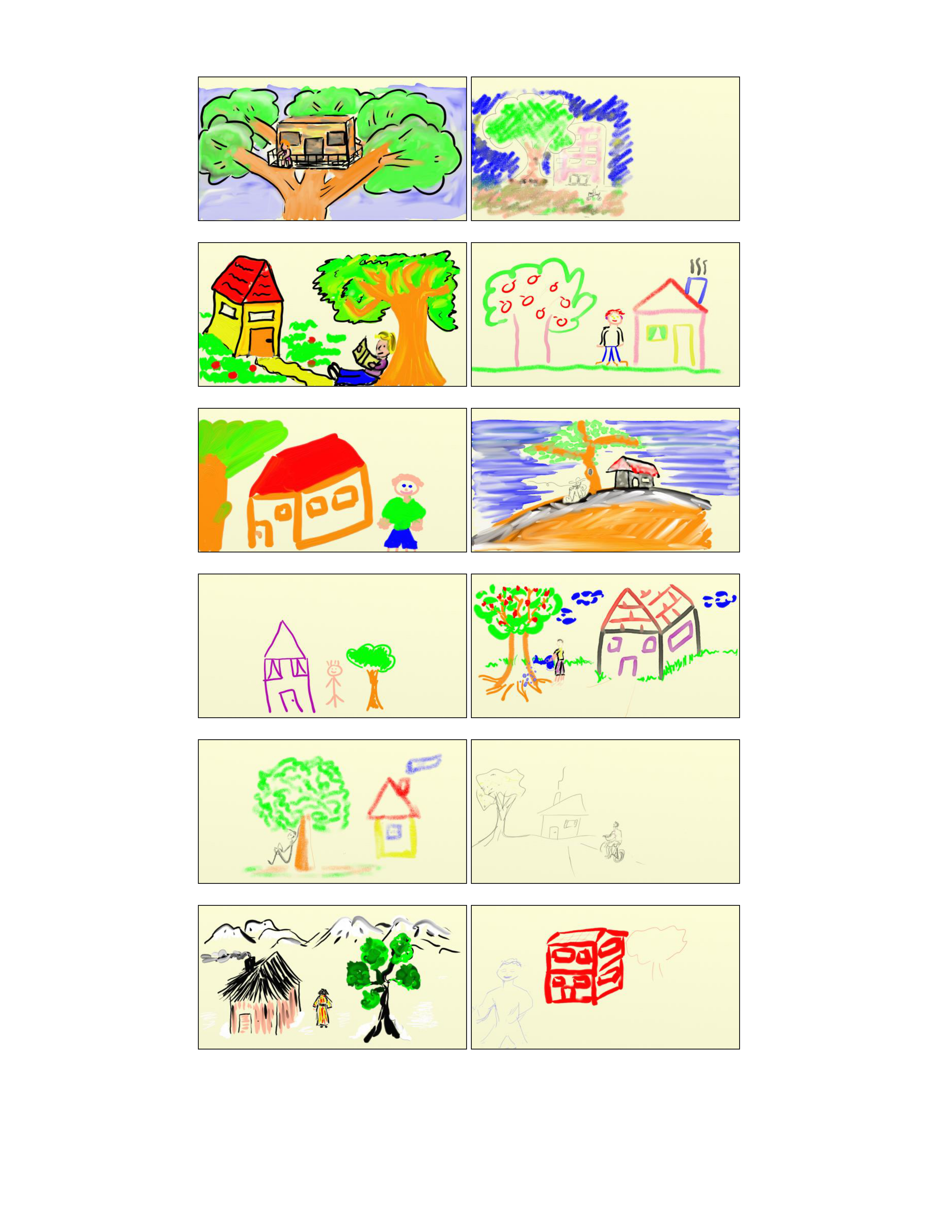

Supplement: S15 Fig — (Left column) Females’ drawings. (Right column) Males’ drawings. (TIF) [file pone.0126467.s015.tif]
